# Vapor Phase Infiltration of Titanium Oxide into P3HT to Create Organic–Inorganic Hybrid Photocatalysts

**DOI:** 10.1021/acsami.3c16469

**Published:** 2024-06-21

**Authors:** Li Zhang, Shawn A. Gregory, Kristina L. Malinowski, Amalie Atassi, Guillaume Freychet, Mark D. Losego

**Affiliations:** † School of Materials Science and Engineering, Georgia Institute of Technology, 771 Ferst Drive NW, Atlanta, Georgia 30332, United States; ‡ Renewable Bioproducts Institute, Georgia Institute of Technology, 500 10th Street NW, Atlanta, Georgia 30332, United States; § NSLS-II, Brookhaven National Laboratory, Upton, New York 11973, United States

**Keywords:** photocatalysis, vapor phase infiltration, atomic layer infiltration, dye-sensitized, conjugated polymers

## Abstract

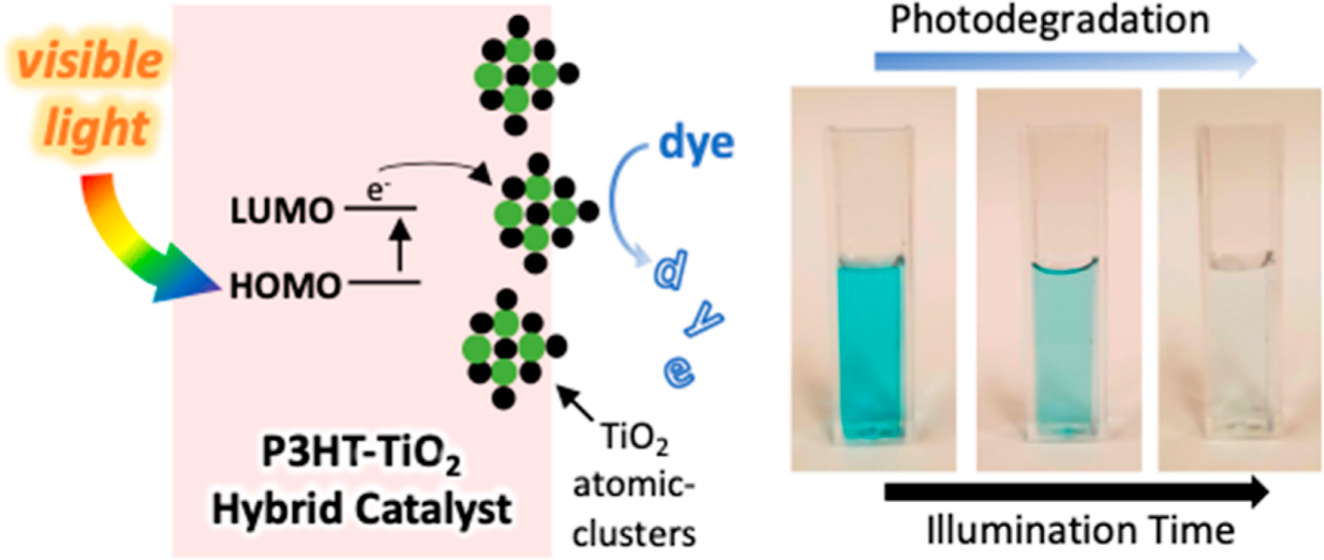

Herein, we report for the first time the use of vapor phase infiltration (VPI) to infuse conducting polymers with inorganic metal oxide clusters that together form a photocatalytic material. While vapor infiltration has previously been used to electrically dope conjugated polymers, this is the first time, to our knowledge, that the resultant hybrid material has been demonstrated to have photocatalytic properties. The system studied is poly(3-hexylthiophene-2,5-diyl) (P3HT) vapor infiltrated with TiCl_4_ and H_2_O to create P3HT-TiO_*x*_ organic–inorganic hybrid photocatalytic materials. X-ray photoelectron spectroscopy analysis shows that P3HT-TiO_*x*_ VPI films consist of a partially oxidized P3HT matrix, and the infiltrated titanium inorganic is in a 4+ oxidation state with mostly oxide coordination. Upon visible light illumination, these P3HT-TiO_*x*_ hybrids degrade methylene blue dye molecules. The P3HT-TiO_*x*_ hybrids are 4.6× more photocatalytically active than either the P3HT or TiO_2_ individually or when sequentially deposited (e.g., P3HT on TiO_2_). On a per surface area basis, these hybrid photocatalysts are comparable or better than other best in class polymer semiconductor photocatalysts. VPI of TiCl_4_ + H_2_O into P3HT makes a unique hybrid structure and idealized photocatalyst architecture by creating nanoscale TiO_*x*_ clusters concentrated toward the surface achieving extremely high catalytic rates. The mechanism for this enhanced photocatalytic rate is understood using photoluminescence spectroscopy, which shows significant quenching of excitons in P3HT-TiO_*x*_ as compared to neat P3HT, indicating that P3HT acts as a photosensitizer for the TiO_*x*_ catalyst sites in the hybrid material. This work introduces a new approach to designing and synthesizing organic–inorganic hybrid photocatalytic materials, with expansive opportunities for further exploration and optimization.

## 1 Introduction

Conjugated polymers (CP) are of interest for flexible electronics,^1^ bioelectronics^2^ and electrochromics,^3^ among other applications.^4^ This interest derives from their mechanical flexibility and ease of engineering optical and electronic properties. To fine-tune the optical and electronic properties of CPs, altering the main chain and/or side chain chemistries, controlling crystallinity, and chemical doping of the polymer are often employed.^5,6^ Of these techniques, chemical doping is widely used because it enables exquisite control over the electronic and optical properties of the CPs. This doping process can be broadly categorized into vapor doping methods (e.g., dopant vapors react with the CP) and solution doping (e.g., solvents swell the CP and dopant solutes react with the CP).^7^ Vapor doping of CP can be advantageous because there is no solvent used, mitigating polymer swelling and alterations to the polymer crystallinity, alignment, conformality, and dimensions.

**Figure 1:**
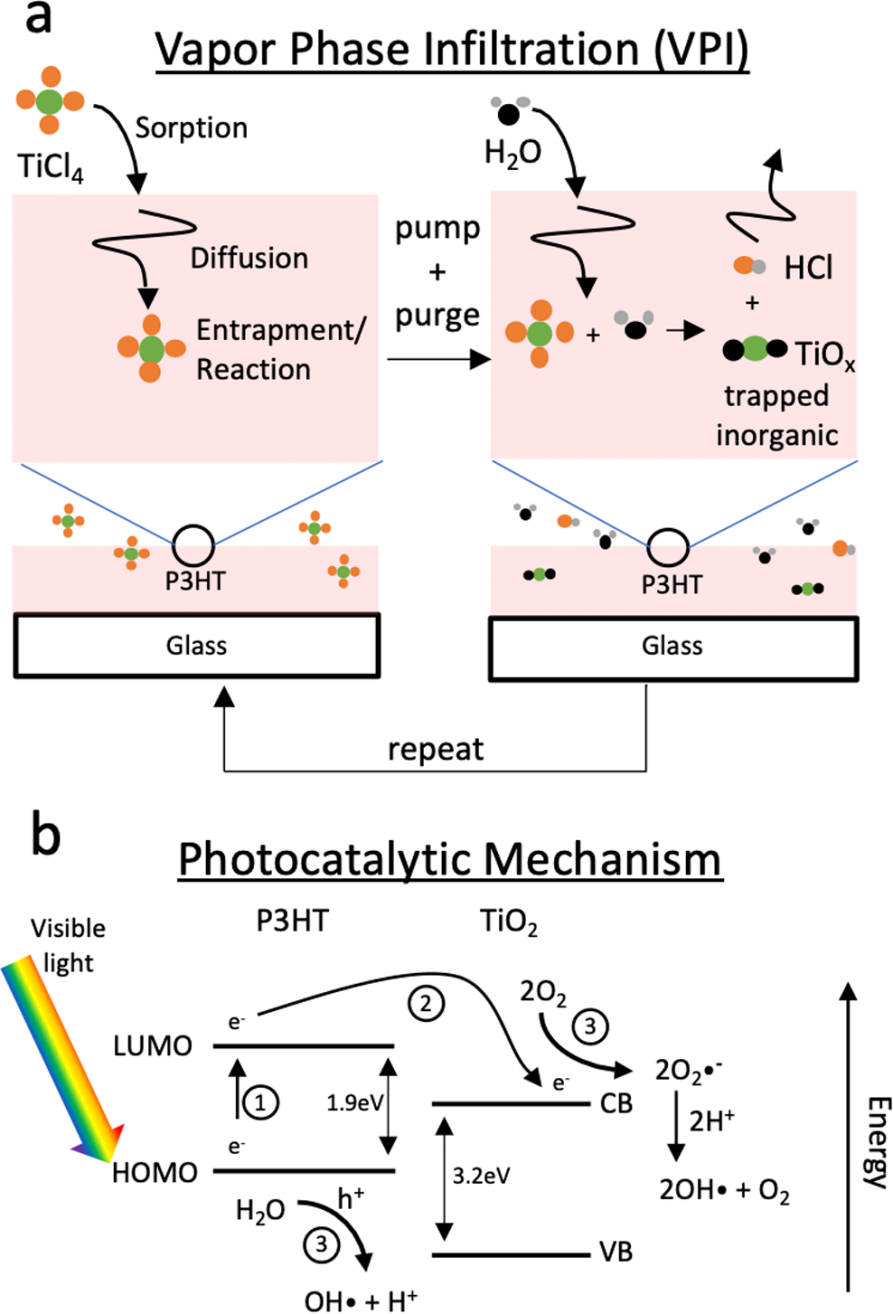
(a) Depiction of a VPI process that includes sorption and bulk diffusion of vapor phase inorganic precursors into the bulk of an organic polymer. (b) Proposed photocatalysis process for a P3HT-TiO_*x*_ system showing (1) visible light exciting an electron from the HOMO to the LUMO in P3HT and leaving a hole behind, (2) injection of the excited electron into the conduction band (CB) of TiO_*x*_, and (3) subsequent reactions of the electron with oxygen and water to create reactive species such as OH radicals that can degrade a variety of compounds.

Several processing methods have been studied for the vapor doping of CPs. One of these approaches is vapor phase infiltration (VPI) with inorganic precursors.^8,9^ VPI is a subtechnique of atomic layer deposition (ALD). However, instead of depositing ultrathin coatings on a substrate, precursors are allowed to diffuse into and react with functional groups in the bulk of the polymer.^10,11^ Traditional ALD processes dose gaseous precursors and coreactants into the reaction chamber separately. The precursor and coreactant independently and sequentially react with the substrate surface in a self-limiting fashion, leading to an atomic-layer-by-atomic-layer deposition of a coating on the substrate’s surface. As depicted in Figure 1a, VPI uses similar gas-phase precursors and coreactants in a sequential dosing manner, but ideally, these precursors are delivered without a carrier gas, and the system is isolated in a static atmosphere of just the precursor gas at a constant pressure for an extended time to permit the sorption and diffusion of these inorganic species into the polymer.^11,12^ The result of VPI, unlike ALD, is a modification of the bulk polymer chemistry, with the entrapment of inorganic clusters, often metal oxides (MO_*x*_) or hydroxides, inside the polymer. These inorganic clusters are seen as detrimental to the overall electronic conductivity of a doped CP because they act as scattering centers that reduce electronic mobility.^8,9,13^ However, here, we demonstrate that these inorganic clusters can actually be utilized as sites for photocatalysis.

One environmentally important application for photocatalysis is dye degradation. Proper degradation of dyes from the textile, paper, and apparel industries is ecologically important because dye-contaminated waters inhibit photosynthesis and increase chemical oxygen demands for ecosystems.^14,15^ Photocatalysts are a promising solution to removing dyes from wastewater streams because they do not produce additional contaminants and may have long lifetimes, leading to favorable economics.^16^ Among the available photocatalysts, TiO_*x*_ has particular promise because of its low toxicity, elemental abundance, and stability.^17,18^

Photoexcitation of an electron from the valence band to the CB enables TiO_*x*_ photocatalysis. The bandgap for TiO_*x*_ is about 3.2 eV, corresponding to a near-ultraviolet wavelength of 387 nm. This UV light makes up only a small fraction of sunlight.^19^ Thus, to make TiO_*x*_ photocatalysts more active in sunlight, photosensitizers are frequently introduced. Photosensitizers that absorb sunlight’s more prevalent visible wavelengths can inject electrons into the CB of TiO_*x*_ if electronic bands are properly aligned. Poly(3-hexylthiophene) (P3HT), with a band gap of about 1.9 eV (652 nm), is well-positioned to perform as a good photosensitizer for TiO_*x*_.

Figure 1b depicts the expected photosensitization process for the P3HT-TiO_*x*_ system. Visible light is absorbed by the CP, creating an exciton that consists of an excited electron and a hole. These photogenerated electrons are injected into the CB of TiO_*x*_, where they can then reduce O_2_ in the presence of water to create reactive species such as OH radicals that serve as disinfectants that degrade a variety of organic compounds, including dyes.^20^ Essential to this photocatalytic process are (1) the photoexcitation of the CP to create an exciton and (2) the subsequent charge transfer of the photoexcited electron from the P3HT to the titania. The exciton diffusion length in P3HT has been reported to be between 3 and 8.5 nm,^21−23^ meaning that TiO_*x*_ must be located no farther than 8.5 nm from where the exciton is generated for charge transfer to occur. A variety of synthesis methods have been employed to make CP-MO_*x*_ composite photocatalysts (e.g., direct mixing of MO_*x*_ and conjugate polymers,^24,25^ coating CP onto MO_*x*_,^26^ and coating MO_*x*_ onto conjugated polymer^27^), all showing the usefulness of this charge transfer to help photosensitize MO_*x*_ for various applications.^19,28−30^ Herein, we use VPI as a method for infiltrating the conjugated polymer P3HT with metal oxide clusters of TiO_*x*_ and then demonstrate the utility of this hybrid material to photocatalyze the degradation of organic dyes.

## 2 Materials and Methods

### 2.1 Film Fabrication and Characterization

#### 2.1.1 Solution Preparation and Spray Coating

Regioregular poly(3-hexylthiophene-2,5-diyl, P3HT, Sigma-Aldrich, molecular weight = 50,000–100,000 g/mol) was dissolved in toluene (Sigma-Aldrich, purity = 99.8%) at 10 mg/mL for thin film fabrication. P3HT films were prepared by spray casting 200 μL of this solution (heated to 50 °C) onto 7.5 cm × 2.5 cm glass slides using a Master Airbrush G22 spray caster. Prior to casting, glass slides were cleaned with isopropyl alcohol and dried with N_2_. After being coated, glass slides were cut into approximately 2.5 cm × 0.7 cm rectangles to fit the catalytic rate measurement setup. Films used for electrical conductivity measurements were cut into approximately 1 cm × 1 cm squares. Film thicknesses were measured by scratching the film with a pair of tweezers and then using a Profilm 3D optical profilometer to measure the step edge.

### 2.2 Vapor Phase Infiltration

VPI was carried out in a custom-built pancake-style reactor using a vertically positioned 6 in. conflat tube (4 in. inner diameter) that is 4 in. in height. The total chamber volume was approximately 50 in.^3^ (820 cm^3^). The top of the chamber has a standard conflating door for sample exchange. The chamber was operated at 80 °C for all of the reactions. A Leybold Trivac D16B with a pump speed of 19.8 m^3^/h was used to evacuate the chamber. Both an activated charcoal and a SodaSorb 9.5 in. VisiTrap Inlet Trap were connected between the reactor chamber and pump in series and are the major sources of flow resistance. The reactor’s valve sequencing was automatically controlled with LABVIEW software using a tree-architecture.^31^

Prior to the reaction, thin films were inserted into the VPI reactor, and the chamber was pumped to its background pressure of 0.01 Torr and then purged with 99.995+% N_2_ for 1000 s at a chamber pressure of 0.8 Torr. The VPI process was started by pumping down for 60 s to background pressure, isolating the chamber, and then opening the valve to the room-temperature TiCl_4_ precursor (Strem Chemicals, 97% purity, Danger: can generate corrosive HCl byproducts) for 1 s to achieve a chamber pressure of ∼4.5 Torr. The P3HT thin films were exposed to the TiCl_4_ precursor for 30 s in a static environment. After this exposure, the chamber was then evacuated down to the background for 60 s. Deionized water was then dosed to a chamber pressure of ∼5.5 Torr and held static for 300 s. Between each VPI cycle, the chamber was evacuated for 30 s, purged with nitrogen to a chamber pressure of ∼0.8 Torr for 60 s, and evacuated down to the baseline for another 60 s. This precursor dose and hold, evacuation, water dose and hold, and vacuum, purge, and vacuum constituted a single reaction cycle, as shown in the inset of Supporting Information Figure S1. The pressure profile of the entire VPI process for a 2 cycle VPI process is shown in Supporting Information Figure S1.

### 2.3 Liquid Doping and Dedoping Procedures

For comparison, P3HT films were also liquid doped with solutions of 50 mM iron(III) *p*-toluenesulfonate hexahydrate (FeTos) in acetonitrile and 50 mM nitrosonium hexafluorophosphate (NOPF_6_) in acetonitrile. FeTos, NOPF_6_, and acetonitrile (anhydrous, purity = 99.8%) were purchased from Sigma-Aldrich. The appropriate doping solution was drop-cast onto the films. Films meant for electrical conductivity measurements received 50 μL, and films meant for catalytic measurements received 150 μL due to their larger surface area. Both were doped for 1 min, followed by rinsing with excess acetonitrile to remove any excess dopant. The films were dried under a vacuum of ∼125 Torr for 10 min to ensure the removal of solvent.

To dedope films, vapor-phase hydrazine exposure was used. Vapor dedoping was chosen to prevent film swelling and changes in crystallinity that occur from exposure to liquid solvents. Hydrazine (35 wt % in water) was purchased from Sigma-Aldrich and was diluted 1:100 using acetonitrile. 200 μL of the solution was transferred into a 20 mL scintillation vial with the film that was to be dedoped. The lid was closed, and the film was exposed to the hydrazine vapors for 30 min. After this hydrazine vapor exposure, the conductivity of the films was too low to be measured by our electrical resistance measurement tool.

### 2.4 Methyl Blue Photodegradation Measurements

To measure the photocatalytic activity, methyl blue (MB) degradation was tracked via its peak UV–vis absorbance. Solutions of MB were made from 20 μL of MB dissolved in 2.5 mL of water. The temporal dependence of the absorbance was used to evaluate photocatalytic degradation kinetics. Prior to collecting rate data, films were pretreated by submersion in MB solution with the same concentration, as previously mentioned, for 1 h in the dark to eliminate any possible nonphotocatalytic reactions or adsorption processes that may occur between the film and MB solution. The film was then immediately placed into a new cuvette containing a fresh MB solution for rate data collection.

To determine the photocatalytic degradation rate, cuvettes containing 2.5 mL of MB solution and the catalyst film were exposed to an OSRAM HALOPAR 16 50 W 120 V light (roughly 350–800 nm, light spectrum shown in Supporting Information Figure S2) and mechanically agitated by attaching the cuvette holder to a vortex mixer. A separate broad-spectrum light source was supplied via a fiber optic light source (the Ocean Insights DH-2000 light source with both a deuterium bulb and a halogen bulb). This light path traveled through the MB solution but did not intersect with the glass slide catalyst. This light beam was detected with a fiber optic spectrometer (Avantes Avaspec-ULS2048CL-EVO-RS detector) to track the optical absorbance of the MB solution over time. An absorbance spectrum was taken every 10 min for 3 h. Despite the pretreatment, some reaction or adsorption behavior was still observed in many cases during the first 10–20 min of measurement. For this reason, the first 30 min was omitted when fitting into the rate law. Specifically, we found photodegradation to follow a first-order rate equation
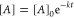
1where *t* is time, [A]_0_ is the initial concentration of MB, [A] is the concentration of MB at time *t*, and *k* is the chemical reaction rate constant. Plots of peak UV–vis absorbance with time were fit to this equation to extract part *k*. In order to correct for differences in the amount of catalyst, glass pieces were weighed, and the effective catalyst area was approximated from the fractional weight of an uncut slide, nominally
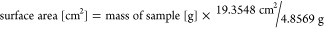
2

The *k*-values were then normalized by dividing the *k*-value by the approximate catalyst surface area. To account for variations in catalytic rate measurements, experiments were performed in triplicate, and the standard deviations are shown as the error bars in the results.

### 2.5 Chemical Characterization and Property Measurement

#### 2.5.1 X-ray Photoelectron Spectroscopy

X-ray photoelectron spectroscopy (XPS) was done using a Thermo Scientific K-alpha system with a monochromatic Al K_α_ X-ray source (1487 eV) having a 60° incident angle and 90° emission collection angle. High-resolution scans were collected at a step size of 0.1 eV. Adventitious carbon (248.8 eV) was used as the charge reference for the analysis. Measurements were taken within 24 h of VPI processing to prevent any dedoping. Depth profiles were performed on the same instrument using a monatomic argon ion gun set at 1000 eV and a medium current for 90 s. At each level, survey scans and elemental analysis were performed.

#### 2.5.2 Reflective Electron Energy Loss Spectroscopy (REELS)

Reflective electron energy loss spectroscopy (REELS) was performed on a Thermo NEXSA G2 XPS system on samples that were not exposed to any X-rays. A pass energy of 10 eV and a step size of 0.1 eV were used. Four scans were collected for each sample.

#### 2.5.3 UV–Vis Spectroscopy

UV–vis spectroscopy was used to characterize the optical absorption of the CP before and after infiltration and doping/dedoping. An Ocean Insights DH-2000 light source and an Avantes Avaspec-ULS2048CL-EVO-RS detector were used for this purpose.

#### 2.5.4 Four-Point Probe

A Keithley 2400 source meter was used to measure the conductivity of the films. A four-point probe head with spring loaded contacts was connected to the source meter. The sheet resistance was measured, and the electrical conductivity was calculated using the film thickness.

#### 2.5.5 Energy Dispersive X-ray Spectroscopy

Elemental analysis was done on samples by using a Phenom ProX benchtop scanning electron microscope (SEM). Energy dispersive X-ray (EDX) spectra were obtained using point analysis while scanning at 15 kV in the backscatter mode for elemental Ti detection. EDX spectra were also obtained in a map analysis at 15 kV in the backscatter mode to show a uniform distribution of Ti throughout the film.

#### 2.5.6 Scanning Electron Microscopy (SEM)

High-magnification images of samples were obtained using a Hitachi SU8230 instrument at 10 kV in the secondary electron mode. A 16.6 mm working distance and a 500,000× magnification was used.

#### 2.5.7 Fourier Transform Infrared Spectroscopy

Functional group analysis was done on samples using a Shimadzu IR Prestige-21 Fourier Transformed Infrared Spectrophotometer in an ATR configuration.

#### 2.5.8 Photoluminescence Spectroscopy (PL)

Analysis of excited electron orbital states was done using a Horiba FL3-21 Fluorometer with a sample angle of ∼30° from the detector. An excitation wavelength of 515 nm was used since it is close to the peak excitation wavelength of P3HT. To account for different optical absorbances between neat and treated P3HT, PL spectra were normalized according to
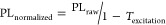
3where PL_normalized_ and PL_raw_ are the photoluminescence of the sample after normalization and as collected, respectively, and *T*_excitation_ is the transmittance of the sample at the excitation wavelength (515 nm in this case).

#### 2.5.9 Grazing Incidence Wide-Angle X-ray Scattering (GIWAXS)

Molecular distance probing was performed at Brookhaven National Lab at the 12-ID Soft Matter Interfaces beamline of National Synchrotron Light Source II (NSLS-II). Polymer films were prepared by spray casting onto p-doped silicon wafers and then treated, as previously described. On each film, 3 measurements were taken at different positions, and the most representative within the series is reported here. A 0.1° angle of incidence was used for all measurements.

## 3 Results and Discussion

### 3.1 Chemical and Electronic Structure of P3HT-TiO_*x*_ Hybrid Films

**Figure 2:**
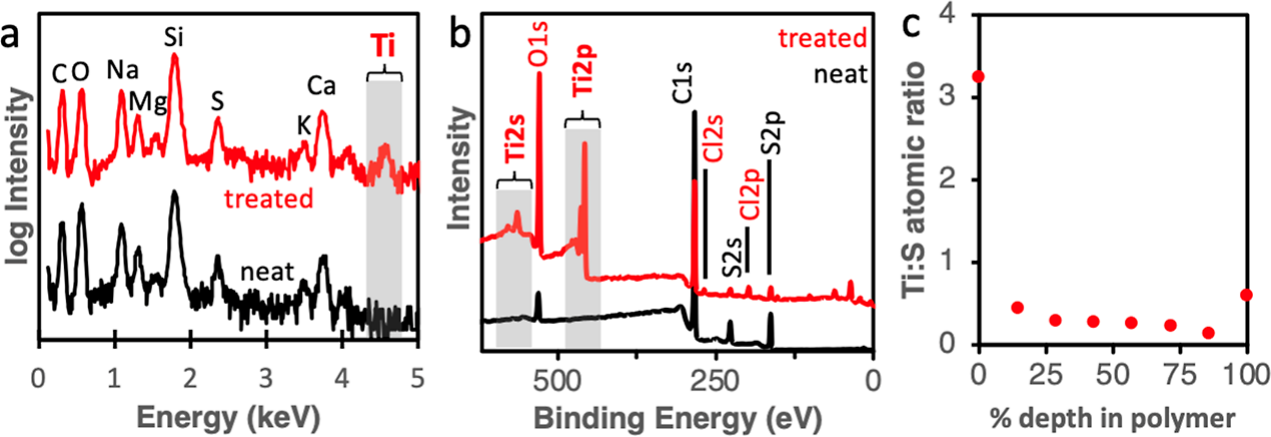
(a) EDX and (b) XPS survey spectra collected from a ∼150 nm neat P3HT film (black, bottom) on glass and a ∼150 nm P3HT film on glass exposed to 5 cycles of TiCl_4_ VPI (red, top). Elements highlighted in red are from the VPI treatment, and elements in black are either inherent to the polymer or part of the glass substrate (Na, Mg, Si, and Ca). (c) XPS depth profile of a ∼150 nm P3HT film exposed to 5 cycles of TiCl_4_ VPI.

VPI of P3HT with metal halides (e.g., MoCl_5_ and FeCl_3_)^8,32^ has been previously reported in the scientific literature, but doping P3HT with TiCl_4_ and H_2_O has not been shown. To confirm that TiCl_4_ and H_2_O infiltrate into P3HT (and to what extent), we first characterized this hybrid material with EDX and XPS. Figure 2 shows both EDX spectra (Figure 2a) and XPS survey spectra (Figure 2b) for a ∼150 nm neat P3HT film and a ∼150 nm P3HT film infiltrated with TiO_*x*_ at 80 °C for 5 cycles. In both Figure 2a,b, the C and S peaks are inherent to the polymer and are labeled in black. In Figure 2a, a clear Ti peak emerges at 4.5 keV in the treated sample, shown in red, that is not present in the neat polymer. Additionally, in Figure 2b, the XPS survey scan further confirms the presence of Ti, with both Ti 2p and Ti 2s peaks present. To quantify the degree of infiltration, an XPS depth profile plotting the Ti/S ratio in the treated polymer is shown in Figure 2c. This depth profile shows a high concentration of Ti near the surface but a lower and somewhat constant concentration within the bulk (Ti/S atomic ratio ≈ 0.3). Based on this Ti/S atomic ratio and reported exciton diffusion lengths, we predict that it is possible for every exciton generated by the P3HT to be quenched by the TiO_*x*_. The calculations to make this prediction are detailed in Supporting Information Section S3 and Figure S3. Additionally, SEM images (Figure S4a) show the inorganic clusters are extremely fine (likely <5 nm in size) and imperceptible by SEM. Additionally, EDX mapping (Figure S4b) shows that the Ti is relatively uniformly distributed throughout the hybrid film. Further details and discussion on SEM images are included in Supporting Information Section S4.

**Figure 3:**
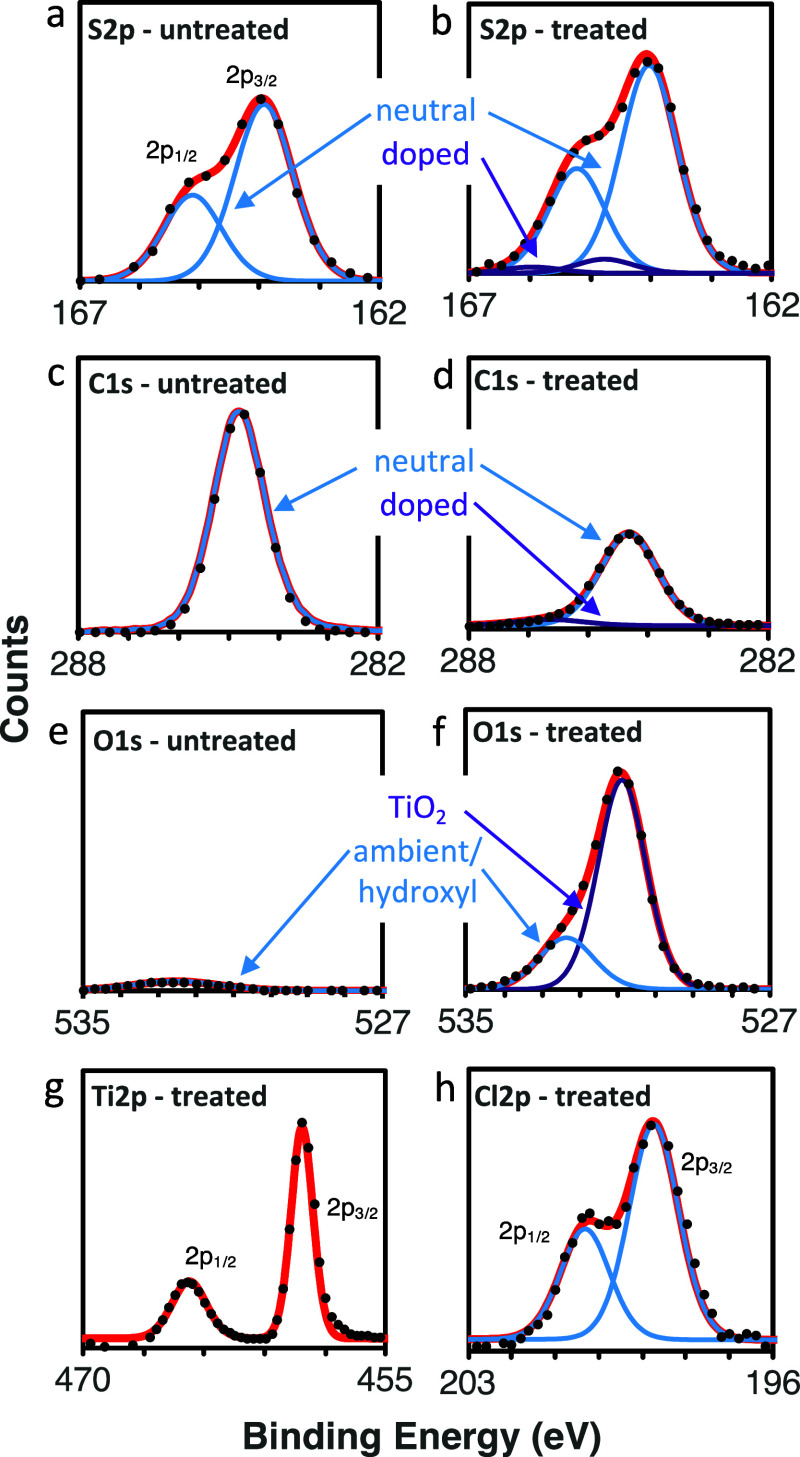
High-resolution XPS spectra of (a,c,e) untreated P3HT films on glass and (b,d,f,g,h) P3HT films on glass exposed to 5 cycles of TiCl_4_ + H_2_O VPI. Included here are XPS elemental spectra for (a,b) S 2p, (c,d) C 1s, (e,f) O 1s, (g) Ti 2p, and (h) Cl 2p with raw data (black points), appropriate deconvolutions (blue and purple), and overall fit (red).

To better understand the P3HT-TiO_*x*_ hybrid structure, high resolution XPS spectra are presented in Figure 3. The C 1s and S 2p spectra are used to characterize the polymer. The neat P3HT S 2p spectrum (Figure 3a) fits well to a single doublet, but the VPI-treated material necessitates a second doublet at higher binding energies (∼0.8 eV higher) for a good fit (Figure 3b). Similarly, the C 1s spectrum for the neat P3HT fits well to a single peak, while the VPI-treated P3HT requires a second peak at higher binding energies to maintain reasonable peak widths and fit (Figure 3c,d). From the literature, it is known that as P3HT becomes doped, the generated polarons create delocalized positive charges that shift the S and C spectra toward higher binding energies.^33,34^ Upon deconvolution, the additional peaks labeled as “doped” in the C 1s and S 2p spectra are consistent with peaks previously observed for polaronic species in doped P3HT. The low intensity of these peaks is consistent with the low amount of doping observed in these materials; the measured conductivity for the P3HT-TiO_*x*_ hybrids is only 2.7 × 10^–6^ S/cm, well below the ∼10° to 10^1^ S/cm values expected for a highly doped P3HT polymer.^35,36^

In Figure 3e–h, the O 1s, Ti 2p and Cl 2p spectra are analyzed to understand the chemical structure of the inorganic. In the untreated P3HT (Figure 3e), minimal oxygen is detected, as expected. After infiltration (Figure 3f), a significant oxygen signal emerges nominally from the infiltrated TiO_*x*_. This oxygen signal can be reasonably deconvoluted into two peaks, one large peak centered at 530.9 eV and a smaller peak at 532.4 eV. These peaks have *fwhm* values of 1.56 and 1.46 eV, respectively, consistent with expectations for O 1s emissions.^37^ The large peak centered at 530.9 eV is consistent with literature reports for metal oxide (M–O–M) chemical states, including TiO_2_. The lower intensity peak at 532.4 eV is more difficult to properly assign. This emission energy is consistent with a variety of C–O bonds,^38^ and matches emissions observed for P3HT oxidized from ambient oxygen.^39,40^ Additionally, some literature reports have found Ti–OH bonds to be near 532 eV with no noticeable changes to the Ti spectrum, but the general instability of titanium hydroxide makes such reports infrequent and somewhat unreliable.^41,42^ To test for the presence of Ti–OH species, we performed FTIR measurements of the hybrid films, and these FTIR do not show any evidence of –OH vibrations (Supporting Information Figure S5). At this point though, it cannot be conclusively determined whether the titanium is not hydroxylated at all or if the concentration of hydroxides is so low that it is undetectable with FTIR.

Next, we turn to interpreting the Ti 2p (Figure 3g) spectrum. A single doublet is observed at energies of 459.2 and 465.38 eV, consistent with the values reported for emission from Ti^4+^ from the 2p_3/2_ and 2p_1/2_ states, respectively.^29,37,43^ The Ti^3+^ oxidation state would have peaks centered at 456.3 and 460.5 eV, respectively; these are not present in our spectrum.^44,45^ Thus, the Ti spectrum provides strong evidence that nearly all of the infiltrated Ti is in the 4+ oxidation state.

Next, we examine the Cl 2p spectrum (Figure 3h). Here, we observe a typical Cl 2p doublet with the 2p_3/2_ emission at 198.7 eV and the 2p_1/2_ at 200.2 eV. These values are within the range for an ionic Cl, which could belong to either a Cl^–^ ion charge balancing a polaron, a trapped H–Cl byproduct formed after reaction between water and TiCl_4_, or an unreacted metal-chloride (Ti–Cl) bond.^9,46,47^ Differentiating among these chemical states is difficult, and unfortunately, the Ti 2p emission for Ti–Cl bonds is nearly the same as that for Ti–O bonds (458.8 eV versus 458.7 eV for the 2p_3/2_ state).^48,49^ From the ALD literature, it is known that at low process temperatures (like those used here), a significant quantity of the TiCl_4_ remains incompletely hydrolyzed, so some amount of TiO_*x*_Cl_4–2*x*_ is possible.^50^ Estimations of the O/Cl atomic percent are 19.5:1, suggesting that if any Ti–Cl exists, it is only at most 5% relative to Ti–O content.

Finally, we want to note that TiO_*x*_S_*y*_ is another potential chemical state for the Ti. However, literature reports for Ti–S place the Ti 2p_3/2_ binding energy near 458.7 eV when 1 > *x*/*y* > 0.2 and multiple 2p_3/2_ peaks are usually observed.^51^ Although the binding energy is within range for what was measured, there is only one peak in the Ti 2p spectrum, and the expected Ti–S bond at around 161 eV in the S 2p spectra is not present.^51,52^ Thus, with the available data, it is reasonable to conclude that no significant amounts of Ti–S bonds are forming.

To investigate the nanoscale structure and impact on P3HT by the TiO_*x*_ clusters made via VPI, GIWAXS measurements were performed (Supporting Information Figures S6–S8), showing that the *d*-spacing between alkyl side chains increases (*q*-spacing decreases), while the *d*-spacing between π–π stacks decreases (*q*-spacing increases) for the infiltrated samples as compared to the neat. Such changes are expected whenever doping occurs in CP, such as P3HT.^53,54^ Further discussion on the diffusion pathway and inorganic localization is in Supporting Information Section S4. In addition to changes in the polymer structure, a new broad peak near 0.2 Å^–1^ is observed in all of the treated samples. This peak increases with VPI cycle count and inorganic abundance and is not present in the neat polymer. The low *q*-spacing (high *d*-spacing) of this peak is indicative of relatively large features. The combination of the emergence of the peak for all treated samples and the low *q*-spacing leads us to speculate that this scattering event emerges from the TiO_*x*_ nanoclusters.

**Figure 4:**
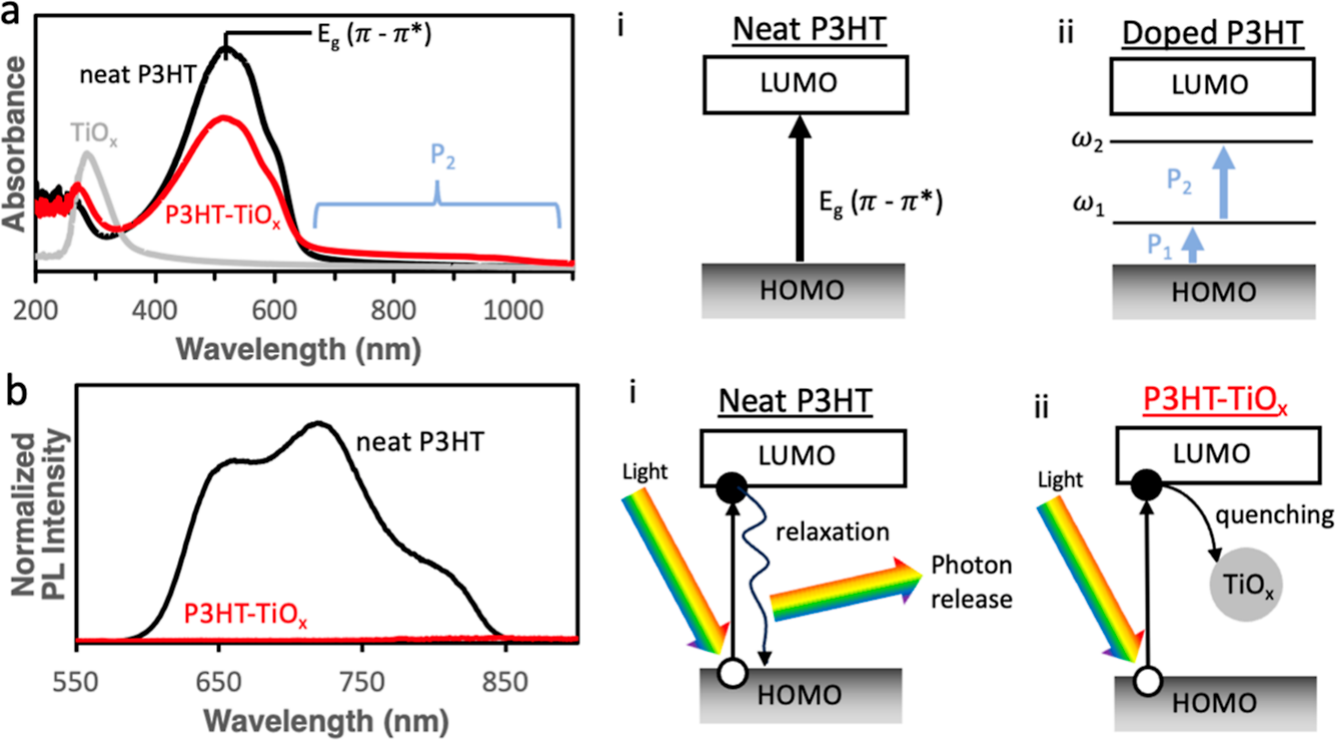
(a) UV–vis spectra for a neat P3HT (black), 200 cycles (∼10.2 nm) of ALD-deposited TiO_2_ (gray), and a P3HT film exposed to 5 VPI cycles of TiCl_4_ + H_2_O (red), all on glass substrates, with (i,ii) relevant electronic band structure for an undoped and doped P3HT. (b) Normalized photoluminescent intensity of neat P3HT (black) and a P3HT film exposed to 5 VPI cycles of TiCl_4_ + H_2_O (red) on glass substrates with (i,ii) depictions of exciton generation and the subsequent relaxation or quenching.

To better understand the electronic structure of these hybrid materials, we use UV–vis–NIR and photoluminescence (PL) spectroscopies. Figure 4a plots the UV–vis absorbance spectra for a 150 nm, undoped P3HT film on glass before (neat) and after 5 cycles of infiltration (P3HT-TiO_*x*_) as well as an ALD deposited TiO_2_ film on glass as a reference. Figure 4a(i,ii) depicts the expected band structures for the undoped and doped P3HT materials. As expected, the pure P3HT absorbs in the visible region (λ_max_ = 517.7 nm), attributed to the π → π* transition [shown in Figure 4a(i)], with near-zero absorbance at wavelengths >650 nm.^55,56^ In contrast, the P3HT-TiO_*x*_ hybrid has a decreased π → π* peak absorbance but higher overall absorbance at longer wavelengths (>650 nm). The reduced π → π* absorbance is indicative of additional states for excitation [e.g., the P1 and P2 transitions indicated in Figure 4a(ii)], reducing the probability for full bandgap excitations.^57^ Similarly, the increased absorbance in the red and near-IR is indicative of a polaronic absorbance in a doped P3HT material and is labeled as the P_2_ transition in Figure 4a(ii).^58^ This emergence of polaron absorbance is consistent with TiCl_4_ oxidatively doping the bulk of the polymer, providing further evidence that an infiltration has occurred. At shorter wavelengths, a strong absorbance emerges in the P3HT-TiO_*x*_ hybrid near 290 nm, similar to the absorbance at 300 nm observed in the TiO_*x*_ ALD film. Literature reports have found that when TiO_*x*_ is mixed with P3HT, the hybrid material will have increased UV absorbance due to the TiO_*x*_ absorbance.^21,59^ The lack of any new absorbance peaks (with the exception of the expected polaronic peak due to doping) indicates minimal interactions of electronic bands between P3HT and TiO_*x*_ in the ground state. REELS measurements performed on the pure and hybrid materials also suggest that the infiltrated TiO_*x*_ clusters maintain a bandgap of about 3.17 eV as expected for amorphous TiO_2_, although the accuracy of this measurement is limited by the low fraction of inorganic in this material (see Supporting Information Figure S9).

To evaluate changes in the electronic band structure in the excited state, photoluminescent (PL) spectra are reported in Figure 4b. These spectra are normalized to the transmittance at the excitation wavelength (515 nm). As depicted in Figure 4b(i), photoexcited electrons in P3HT are expected to decay with the release of a photon in PL, giving the emission spectrum observed in black in Figure 4b. If these photoexcited electrons are successfully injected into the infiltrated TiO_*x*_ clusters in the hybrid material, then this photoemission should be quenched, as depicted in Figure 4b(ii). Indeed, as shown in Figure 4b, the PL spectrum for the P3HT-TiO_*x*_ hybrid exhibits a 2 to 3 orders of magnitude decrease in PL intensity. Possible quenching mechanisms are further discussed in Supporting Information Section S5. Regardless of the exact quenching process, this reduction in PL intensity strongly suggests that the photoexcited electrons in the P3HT are being injected into TiO_*x*_ and will be available for photocatalysis.^22,60−62^

### 3.2 Photocatalytic Performance

**Figure 5:**
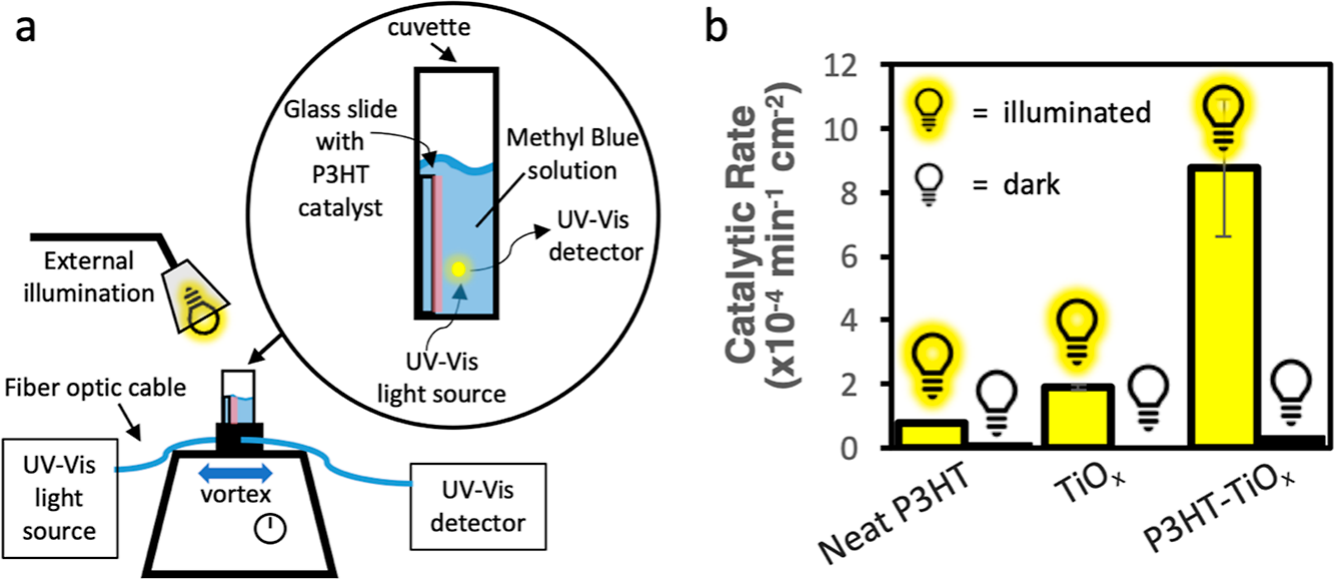
(a) Depiction of the experimental setup used to obtain catalytic rates using degradation of methylene blue dye tracked via UV–vis spectroscopy. (b) Reaction rate constants for degradation of MB using neat P3HT films on glass, 50 cycles of ALD-deposited TiO_2_ films on glass, and P3HT films on glass exposed to 5 cycles of TiO_*x*_ VPI. Measurements made under illumination are shown in yellow on the left, and those made in the dark are shown in black on the right.

Figure 5a depicts the experimental setup used to measure photocatalytic activity and the two light sources used to make measurements. Notably, one light source is used to measure the absorbance of the MB solution and does not interact with the thin film photocatalyst, while the other light source is a broad band light (350 to 800 nm) specifically used to photoactivate the thin film photocatalyst. In the Supporting Information Section S6, an example calculation using Figure S10 shows the experimental methods and equations used to obtain the photocatalytic rate constant. Prior to any measurement for the photocatalytic rate, samples were submerged in MB solution under dark conditions for 1 h to allow for any noncatalytic processes (reaction with HCl byproduct, adsorption, etc.) to occur.

Figure 5b plots the area-normalized rate constants for photocatalytic degradation of MB measured for neat (undoped) P3HT, ALD-deposited TiO_2_, and VPI-synthesized P3HT-TiO_*x*_ films under illuminated and dark conditions. All materials show more photocatalytic activity when illuminated than in the dark. Compared to the controls shown here, the P3HT-TiO_*x*_ has significantly higher photocatalytic reactivity, about 11× higher than neat P3HT and 4.6× higher than pure TiO_2_. This result confirms that the hybrid exhibits a light-activated synergistic photocatalytic effect between P3HT and TiO_*x*_ that exceeds the photocatalytic performance of either component individually. Illumination is clearly necessary to activate this response, as the catalytic degradation rate for the P3HT-TiO_*x*_ hybrid in the dark is near zero, while once illuminated, it exceeds 8 × 10^–4^ min^–1^ cm^–2^. This necessity for light and the combination of both P3HT and TiO_*x*_ components provide evidence that this is a synergistic phenomenon, with P3HT likely acting as a sensitizer for TiO_*x*_, consistent with the mechanism of Figure 1b and PL measurements from Figure 4b.

**Figure 6:**
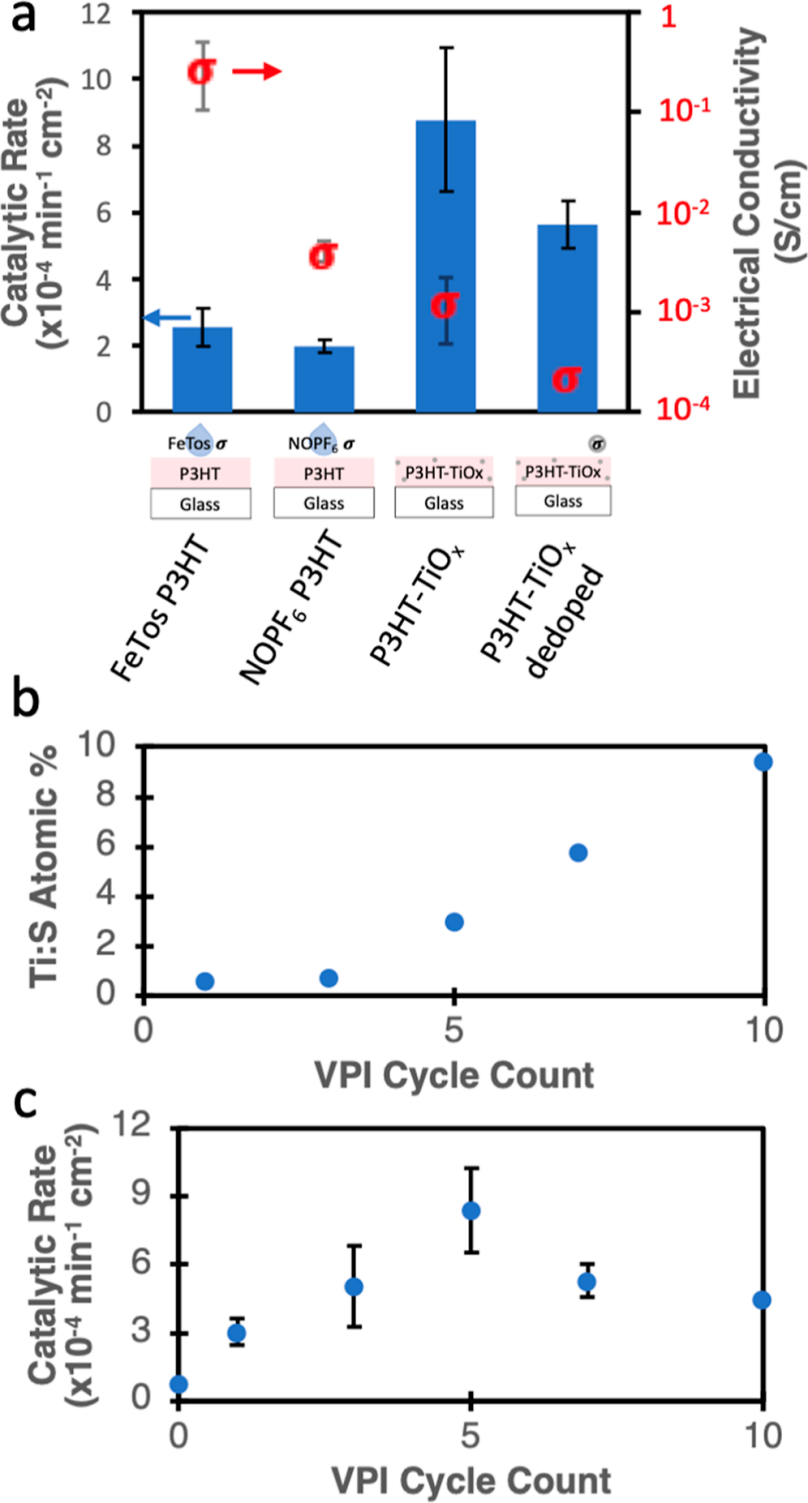
(a) Photocatalytic rates (left *y*-axis) and electrical conductivities (right *y*-axis) of P3HT films on glass treated with FeTos and NOPF_6_ liquid doping, 5 cycles of VPI, and 5 cycles of VPI, then hydrazine vapor dedoped. Note: conductivity of the dedoped sample was unmeasurable but is displayed as the lower limit of detectability for the measurement device. (b) Ti/S atomic % determined by XPS and (c) catalytic rate for P3HT films on glass exposed to a variety of VPI cycles.

Notably, this TiCl_4_ VPI process also dopes/oxidizes P3HT and increases the electrical conductivity, which is not a mechanism present in other physically blended CP-MO_*x*_ composites. To understand the effects of semiconducting polymer doping and electrical conductivity on photocatalytic activity, a series of control systems are investigated. Figure 6a shows both the electrical conductivities of these films (red dots, right axis) and the photocatalytic reaction rates (blue bars, left axis). Specifically, we test: (1) P3HT liquid doped with FeTos (contains Fe inorganic), (2) P3HT liquid doped with NOPF_6_ (contains no metals), (3) VPI synthesized P3HT-TiO_*x*_ hybrids (same hybrid data as in Figure 5b), and (4) VPI synthesized P3HT-TiO_*x*_ that has been dedoped with hydrazine vapor. As shown in Figure 6a, the FeTos and NOPF_6_ films, are significantly more electrically conductive than the P3HT-TiO_*x*_ hybrids (5× to 100× more conductive). While these liquid-doped systems exhibit higher photocatalytic reaction rates than undoped P3HT, they are still 3–5× lower in photocatalytic activity than the P3HT-TiO_*x*_ hybrid. While dedoping of the hybrid does lower its photocatalytic activity, these dedoped P3HT-TiO_*x*_ films remain more photocatalytically active than either FeTos or NOPF_6_-doped pure CP. These results agree with Xu et al., who reported an optimal oxidation of P3HT to achieve the highest photocatalytic activity in P3HT-metal oxide nanocomposites.^63^ Ultimately, these results suggest that the presence of the inorganic TiO_*x*_, not doping or electrical conductivity alone, is the primary driver for enhanced photocatalytic activity in this system.

Note that the XPS depth profiles (Figures 2c and S3) indicate that the infiltrated inorganic is more concentrated near the surface of the hybrid film, and thus, it is likely that the film’s electrical conductivity varies with depth. Thus, the conductivity values reported in Figure 6a are likely an “average” conductivity for the hybrid films. However, the multiple orders of magnitude differences between the P3HT-TiO_*x*_ hybrid films and the liquid-doped films still provide strong evidence that the observed changes are not driven by conductivity alone but rather by the synergistic presence of the inorganic.

Prior synthesis methods of CP-MO_*x*_ photocatalysts have also shown that the amount of MO_*x*_ affects the photocatalytic activity.^19,64^ In order to study this effect, a series of P3HT-TiO_*x*_ hybrids were prepared in increasing numbers of VPI cycles. Figure 6b plots the Ti/S ratio measured via XPS for each of these conditions. This data demonstrates that Ti concentration (at least at the near surface) increases with the number of cycles. Figure 6c plots the corresponding photocatalytic degradation rates as a function of the number of VPI cycles. Here, the photocatalytic degradation rate peaks at 5 VPI cycles, which equates to a Ti/S surface ratio of 3. This result indicates that the photocatalytic activity does not continue to increase with increasing inorganic concentrations. We postulate two possible explanations for this behavior. First, we speculate that the charge transport between the P3HT and surface TiO_2_ may be hindered as the TiO_*x*_ volume increases, limiting injection efficiency. Second, as more cycles are applied, the clusters may begin to coalesce into a more continuous film that effectively decreases the effective surface area of the TiO_*x*_ catalyst. However, more studies are needed to fully understand this phenomenon.

### 3.3 Comparison to Prior Reports

**Figure 7:**
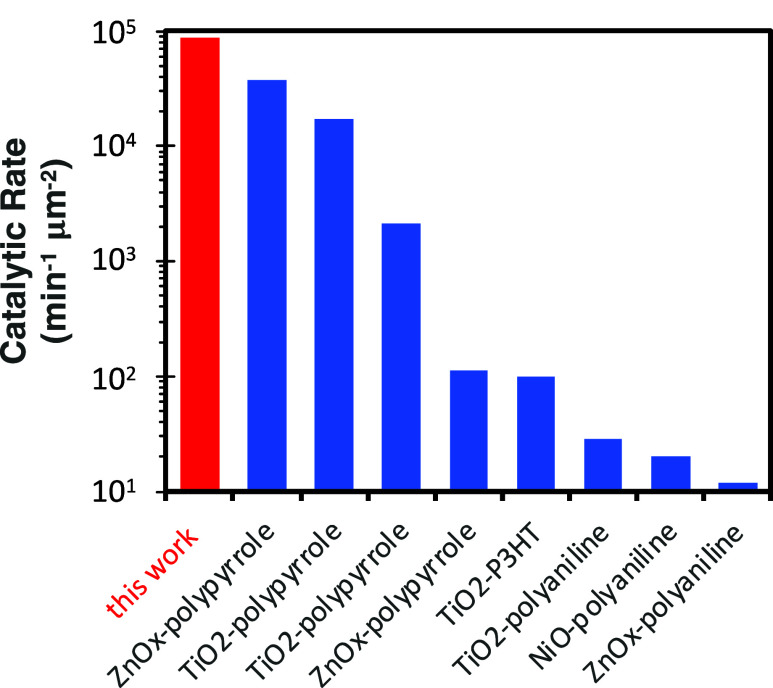
Comparison of other conjugated polymer–metal oxide photocatalysts used for dye degradation from the literature. Note: many studies were excluded from this comparison if the surface area for the catalyst could not be easily/confidently calculated.

To better contextualize the results presented herein, we compare the VPI P3HT-TiO_*x*_ hybrid catalysts to those of other CP-MO_*x*_ photocatalysts reported in the literature. This comparison is presented in Figure 7 with Table S1, which includes some notes about calculations made and references for each data point. Note that accurately normalizing catalytic rates is difficult given the variation in experimental methods used (e.g., catalyst loading, concentration of MB, light intensity, etc.), but this table provides reasonable insights into how our catalyst generally compares after normalizing to the reported catalyst surface area. Our best performing photocatalyst has a reaction rate constant of 8.7 × 10^–4^, which is comparable to the highest performing CP-MO_*x*_ photocatalysts reported in the literature.

Additionally, some general trends can also be highlighted in this comparison. A majority of the CP-MO_*x*_ photocatalysis publications report the effects of the MO_*x*_/CP ratio. In most of these studies, the catalytic rate initially increases and then decreases with increasing MO_*x*_/CP, similar to our observations.^19,64^ The reason for the initial increase could simply be the increasing surface area of MO_*x*_, which appears to be the primary site for catalytic reactions to occur. Eventually, however, the photocatalyst may be limited by the amount of excitons able to reach the metal oxide catalyst sites.

Many of the lower-performing photocatalysts have been made by coating CPs onto metal oxide particles^19,25,64^ or synthesizing the CP with the MO_*x*_ in solution in a large excess.^65^ These catalyst architectures would bury the metal oxide surface below the CP, making the catalytic sites less accessible to the chemicals being degraded. Conversely, many of the highest performing photocatalysts use thin CP coatings on a metal oxide^66^ or simply have the metal oxide exposed (our work). Based on our understanding of the CP-MO_*x*_ photocatalytic mechanism, Figure S11 presents various catalyst designs and their respective benefits and drawbacks. Based on our XPS depth profiles, VPI appears to generate a hybrid structure in which the metal oxide clusters are at or near the surface of the CP, thus achieving a design similar to that in Figure S11e. To test the merit of this design, we sought to produce several other designs that are expected to be less optimal.

Figure S12 presents the results of these different designs. Specifically, we tested (1) 50-cycles of ALD-deposited TiO_2_ recoated with neat P3HT and (2) P3HT exposed to 5 cycles of TiCl_4_ + H_2_O VPI (our nominal catalyst) recoated with neat P3HT. These are compared to prior data for a pure TiO_*x*_ ALD film and the 5 cycle VPI P3HT-TiO_*x*_ catalyst. Figure S12 clearly shows that both tests are less photocatalytic than the VPI-synthesized P3HT-TiO_*x*_ and less photocatalytic than the ALD-deposited TiO_2_ film. These results provide further evidence that a P3HT surface layer impedes overall photocatalytic performance because the metal oxide is no longer exposed to the reactant species. This requirement sets VPI apart as an effective method to achieve near-surface metal oxide sites, creating superior catalyst architectures. This difference in catalyst architecture is important to note; while prior CP-MO_*x*_ photocatalysts have used similar chemistries (P3HT + TiO_2_), the distributions have been more akin to those of composites or nanocomposites. Here, we have atomic-scale clusters of titanium oxide mixed intimately within the P3HT chains. Likely, both this more intimate intermixing of organic and inorganic materials (which should facilitate photoelectron injection) and the localization of TiO_*x*_ near the catalyst reaction surface (which makes interaction with the target dye molecules more direct) are contributing to the higher reactivity of this hybrid catalyst.

Finally, it is worth mentioning that highly effective photocatalysts are photostable and often nanostructured to increase the effective surface area. In Supporting Information Section S9, the photostability of our hybrid catalyst is tested. No change is observed in the FTIR and UV–vis spectra before and after a 4 h water submersion under illumination (Figure S13a,b). Additionally, consecutive photocatalytic tests with the same sample showed that the catalyst can be reused and is recyclable (Figure S13c). While P3HT will likely exhibit long-term stability issues, more aqueous-stable CP has been demonstrated and could be of interest for the future development of hybrid photocatalysts.^67^ We further discuss the stability and limitations of the available technology in Supporting Information Section S9. As for nanostructuring, we have chosen to focus on purely 2D P3HT films in this article because of their simplicity in physical and chemical characterization. However, it is likely that much higher degradation rates can be achieved from these VPI P3HT-MO_*x*_ hybrid photocatalysts if a nanostructured surface is used. Work has been done to attach P3HT polymer chains to nanoparticles^68^ as well as make self-supported P3HT nanoparticles,^69^ so this opens the possibility for much higher-performing designs.

## 4 Conclusions

This work demonstrates that the VPI of P3HT with TiCl_4_ and H_2_O can be used to synthesize an organic–inorganic hybrid photocatalyst material. XPS shows that the infiltrated inorganic primarily exists as oxidized titania and that the conjugated polymer is doped during the VPI process. The hybrid material is significantly more photocatalytically active than either the polymer or metal oxide individually, but only when illuminated. Combined with the PL quenching observed for the P3HT-TiO_*x*_ hybrids, these results provide strong evidence that P3HT is acting as a good photosensitizer for the infiltrated TiO_*x*_ inorganics. Through doping and dedoping studies, we show that higher electrical conductivity alone is not sufficient to explain the observed photocatalytic enhancement, but rather that the presence of the TiO_*x*_ inorganic is essential. When comparing the hybrid material made by VPI to other CP-MO_*x*_ photocatalysts, good catalyst architecture design seems to include keeping the MO_*x*_ catalyst near the reactive surface, as accomplished here. Control studies reaffirm that having the metal oxide near the CP’s surface, as is the nature of VPI, is critical to achieving the highest photocatalytic activities. In total, these results introduce a new approach for creating high-performing organic–inorganic hybrid materials for photocatalytic degradation of chemical contaminants.

## Supporting Information Available

Pressure profile for VPI, light spectrum of illumination bulb, discussion on exciton diffusion length and quenching, additional FTIR, GIWAXS, and REELS physical/chemical characterization, exciton quenching mechanism, details on catalysis measurement, discussion on catalyst architecture considerations, notes about the works compared in Figure 7, and discussion and data on catalyst stability (PDF)
