# Supplementary material for: Vapor Phase Infiltration of Titanium Oxide into P3HT to Create Organic–Inorganic Hybrid Photocatalysts
Source: ACS Appl Mater Interfaces. 2024 Jun 21;16(26):33259–69. doi: 10.1021/acsami.3c16469 (PMC11231981; doi:10.1021/acsami.3c16469)
Supplement: Supplementary file 1 — am3c16469_si_001.pdf [file am3c16469_si_001.pdf]

Supporting Information

for

Vapor Phase Infiltration of Titanium Oxide into P3HT to Create Organic-Inorganic Hybrid Photocatalysts

Li Zhang[a,b], Shawn A. Gregory[a], Kristina Malinowski[a], Amalie Atassi[a], Guillaume Freychet[c], Mark D. Losego[a,b,]*

[a]School of Materials Science and Engineering, Georgia Institute of Technology, 771 Ferst Drive NW, Atlanta, GA 30332, USA

[b]Renewable Bioproducts Institute, Georgia Institute of Technology, 500 10th St. NW, Atlanta, GA, 30332, USA

[c]NSLS-II, Brookhaven National Laboratory, Upton, New York 11973, United States

[d]Contact: losego@gatech.edu

Contents

S1. Pressure Plot of Vapor Phase Infiltration Run …………………………………………….…… S-3
S2. Light Spectrum of Illuminating Bulb …………………………….……………….……………. S-4
S3. Potential Exciton Diffusion Length and Quenching..…………….………………………... S-5
S4. Additional P3HT-TiOx Physical/Chemical Characterization …………………………….. S-6
S5. Exciton Quenching Mechanism …………………………………………….……………. S-13
S6. Measuring Photocatalytic Performance ………………………..…….………...……. S-13
S7. Catalyst Architecture Considerations ……………………………..…………...…. S-15
S8. Comparison to Prior Reports …………………………………………….……..…… S-17
S9. Hybrid Material Stability …………………………………………………………….S-18
References ……………………………………………………………………..……..…….. S-20

S1. Pressure Plot of Vapor Phase Infiltration Run

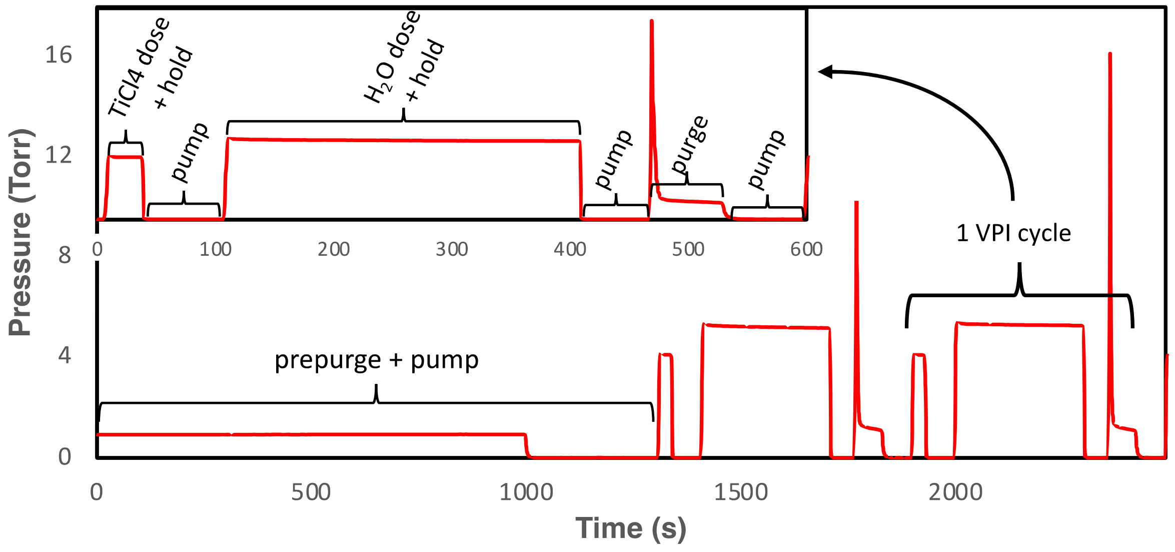

Figure S1. *Plot of in situ pressure profile for an entire VPI process for a 2 cycle exposure with inset showing the $TiCl_4$ dose and 30 s hold; 60 s vacuum; H_2O dose and 5 min hold; and, finally, 30 s vacuum, 60 s purge and 60 s vacuum for an individual cycle.*

Figure S1 shows the pressure profile for an entire 2 cycle VPI run. Note the 1000s prepurge and 300s evacuation to remove any adsorbed water/contaminants that may have entered the chamber when loading samples.

S2. Light Spectrum of Illuminating Bulb

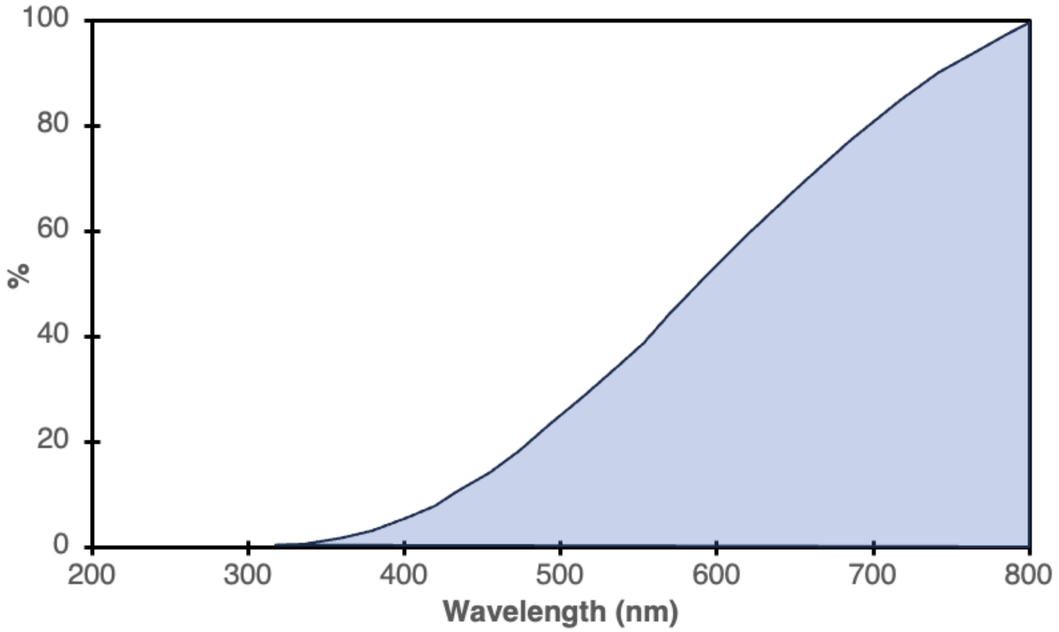

Figure S2. *Light spectrum of OSRAM HALOPAR 16 50 W 120V external bulb used in photocatalysis measurements; this spectrum is adapted from the manufacturer (Osram) website.*

Figure S2 shows the light spectrum of the light bulb used to illuminate the thin film sample during photocatalytic experiments. This spectrum was made publicly available by Osram.

S3. Potential Exciton Diffusion Length and Quenching

For the exciton transfer from the P3HT to the TiO_x, there must be a TiO_x cluster available to absorb the excited electron before the exciton recombines. The average distance an exciton is able to travel before recombining is defined as the exciton diffusion length. For P3HT, the exciton diffusion length has been measured to be around ~3 to 8.5 nm (~7.5 to 21 monomers).[1] Using the XPS depth profiles we can estimate the distance between Ti clusters. As shown in Figure 2C, the Ti:S ratio is ~0.3, equating to a Ti for every 3 monomers, although this assumes a uniform distribution of Ti and no clustering, well within the reported exciton diffusion range. To reduce any potential inaccuracies due to clustering of the Ti in the polymer, which is quite likely given the multiple cycles treatment, we took depth profiles of a 1 cycle infiltration sample (Figure S3) and found a Ti:S ≈ 0.1 in the bulk of the polymer. This corresponds to a Ti for every 10 monomers, slightly higher than the lower limit of the exciton diffusion range. At these concentrations, the TiO_x clusters theoretically should be sufficient to collect nearly every exciton generated by the P3HT.

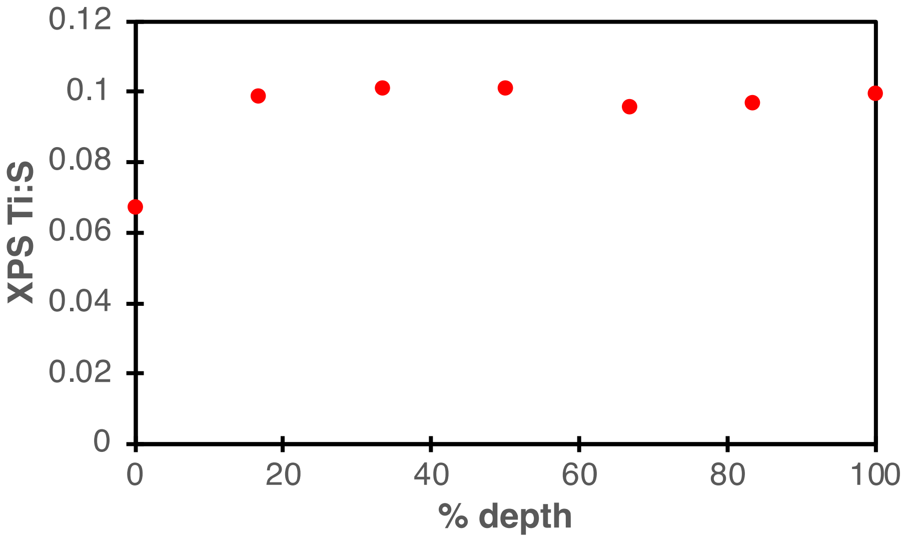

Figure S3. *XPS depth profile of a ~150 nm P3HT film on glass exposed to 1 cycle of $TiCl_4$ VPI.*

S4. Additional P3HT-TiO_x Physical/Chemical Characterization

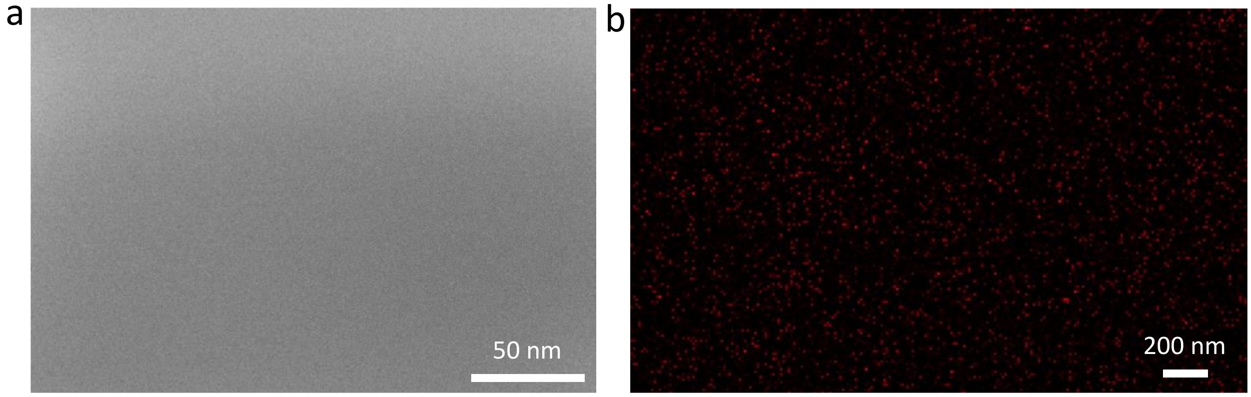

Figure S4. *(a) SEM image and (b) EDX mapping of P3HT exposed to 5 VPI cycles of TiO_x.*

SEM image (Figure S4a) shows that the inorganic clusters infiltrated during VPI cannot be seen in the hybrid material. EDX mapping (Figure S4b) shows a homogenous distribution of Ti throughout the plane of the polymer film. The inability to image the infiltrated clusters is expected as this has been observed in previous publications as well.[2] Additionally, ALD studies have shown $TiCl_4$ + H_2O deposition to result in ~0.05 nm/cycle.[3] This would equate to a theoretical max of 0.45 nm clusters, below the practical resolution of SEM imaging.

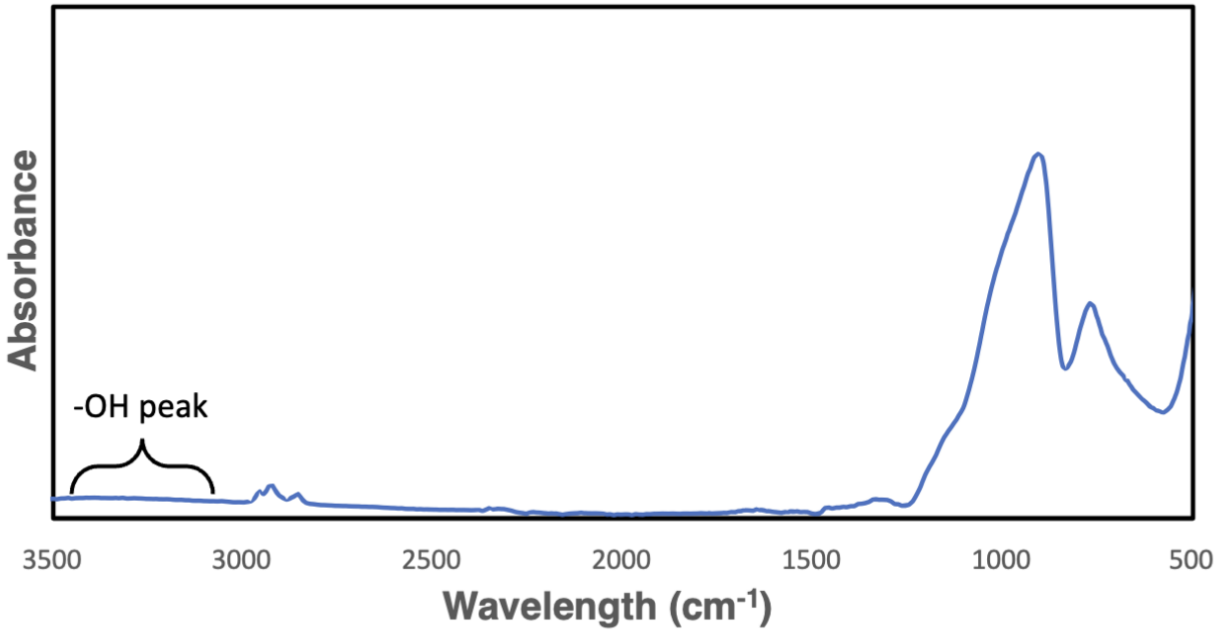

Figure S5. *Fourier Transform Infrared Spectroscopy on P3HT-TiO_x films synthesized using 5 cycles of VPI.*

FTIR spectra were obtained in hopes of determining if Ti-OH bonds were present within the hybrid. The FTIR spectra (Figure S5) does not have the broad hydroxyl peak around 3300cm^{-1}. We are unable to say if this is because there are no hydroxyl groups present or if it is because there are not enough hydroxyl groups present.

Horizontal integration/in-plane orientation

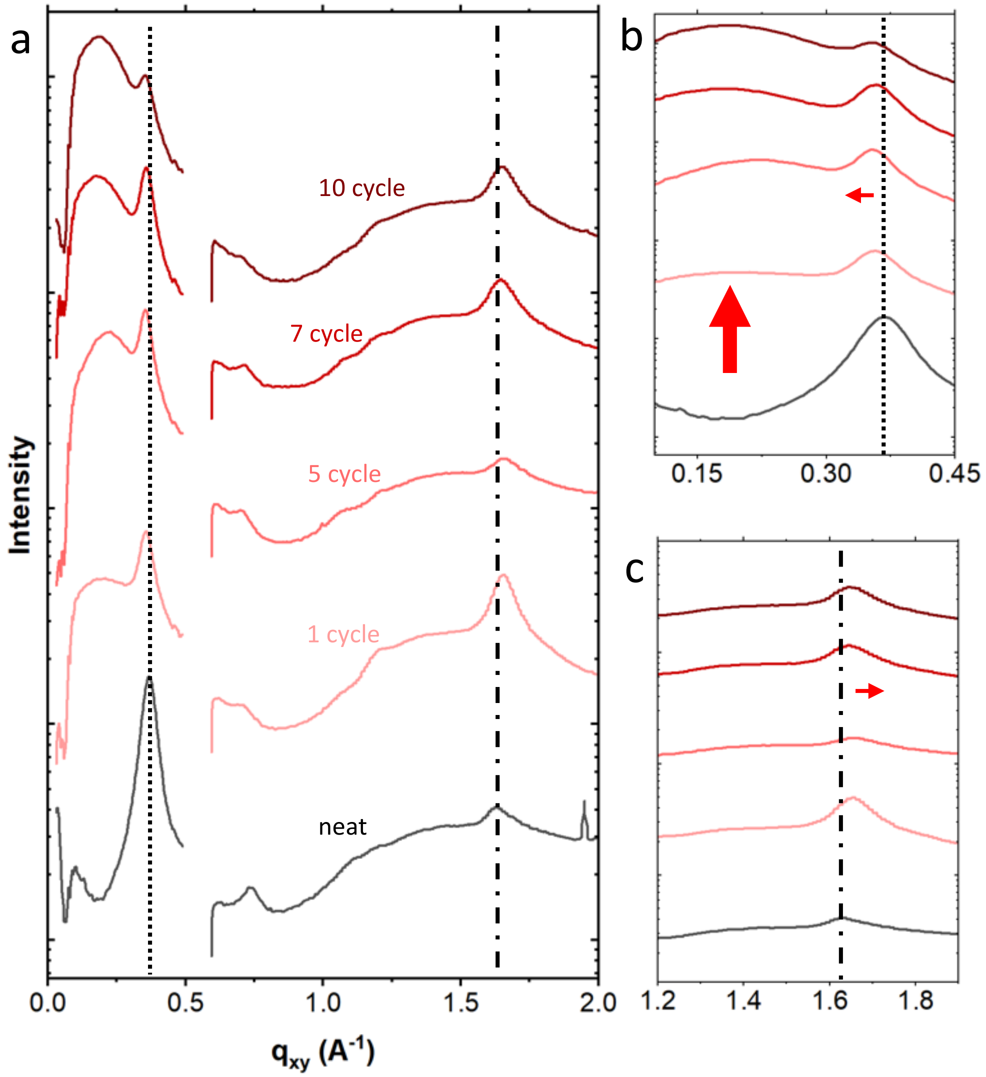

Figure S6. *GIWAXS in-plane line cuts for neat P3HT and P3HT exposed to 1, 5 ,7 and 10 cycles spray casted onto P-doped silicon wafers. (b) and (c) are insets of (a) in the range of 0.1-0.45 and 1.2-1.9 Å^{-1}, respectively, used to highlight the peak shifts from neat to the treated samples as a collective with vertical dashed line and arrows as a guide.*

Vertical integration/nearly out-of-plane orientation

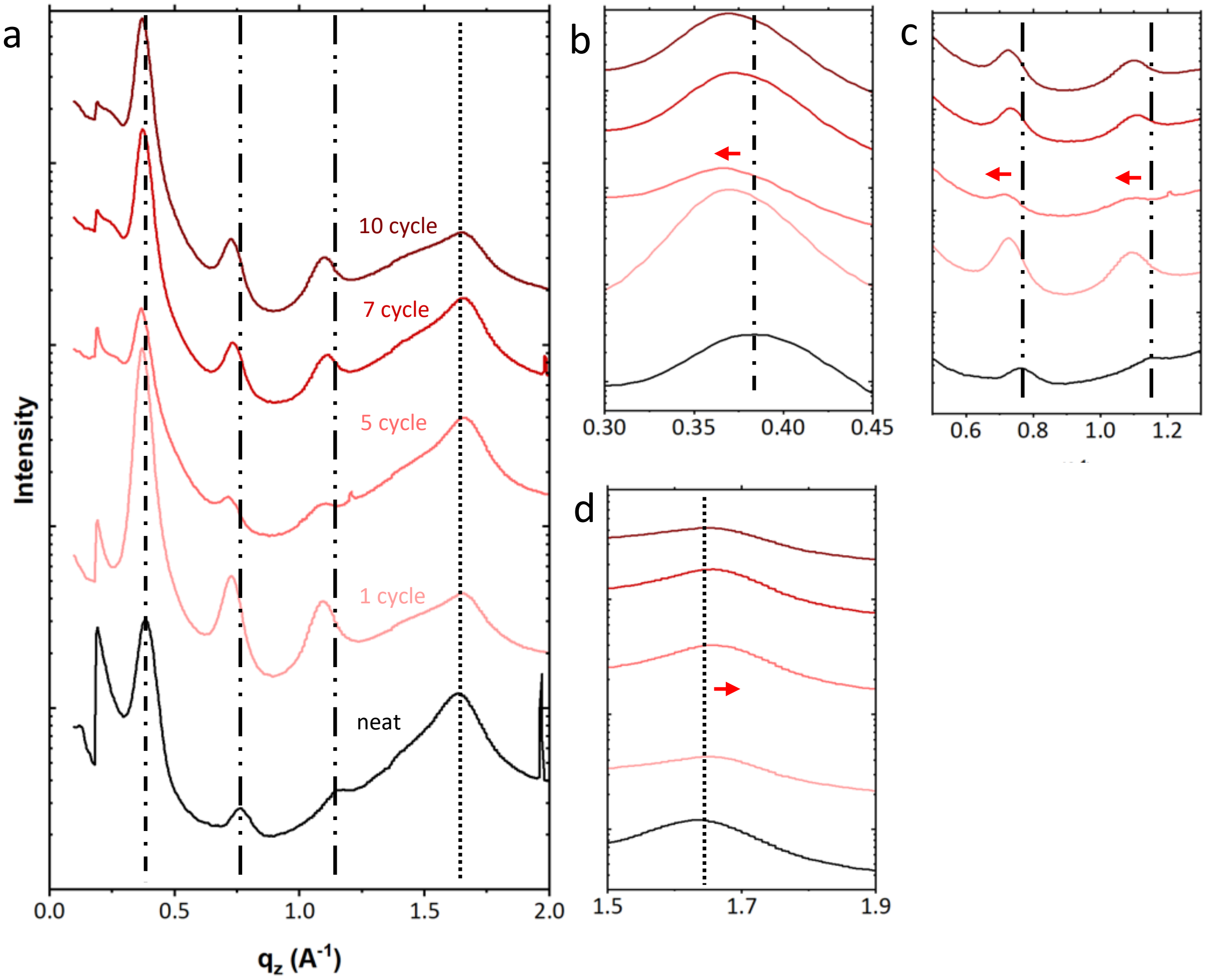

Figure S7. *GIWAXS nearly out-of-plane line cuts for neat P3HT and P3HT exposed to 1, 5 ,7 and 10 cycles spray casted onto P-doped silicon wafers. (b), (c) and (d) are insets of (a) in the range of 0.3-0.45, 0.5-1.3 and 1.5-1.9 Å^{-1}, respectively, used to highlight the peak shifts from neat to the treated samples as a collective with vertical dashed line and arrows as a guide.*

GIWAXS Diffractograms

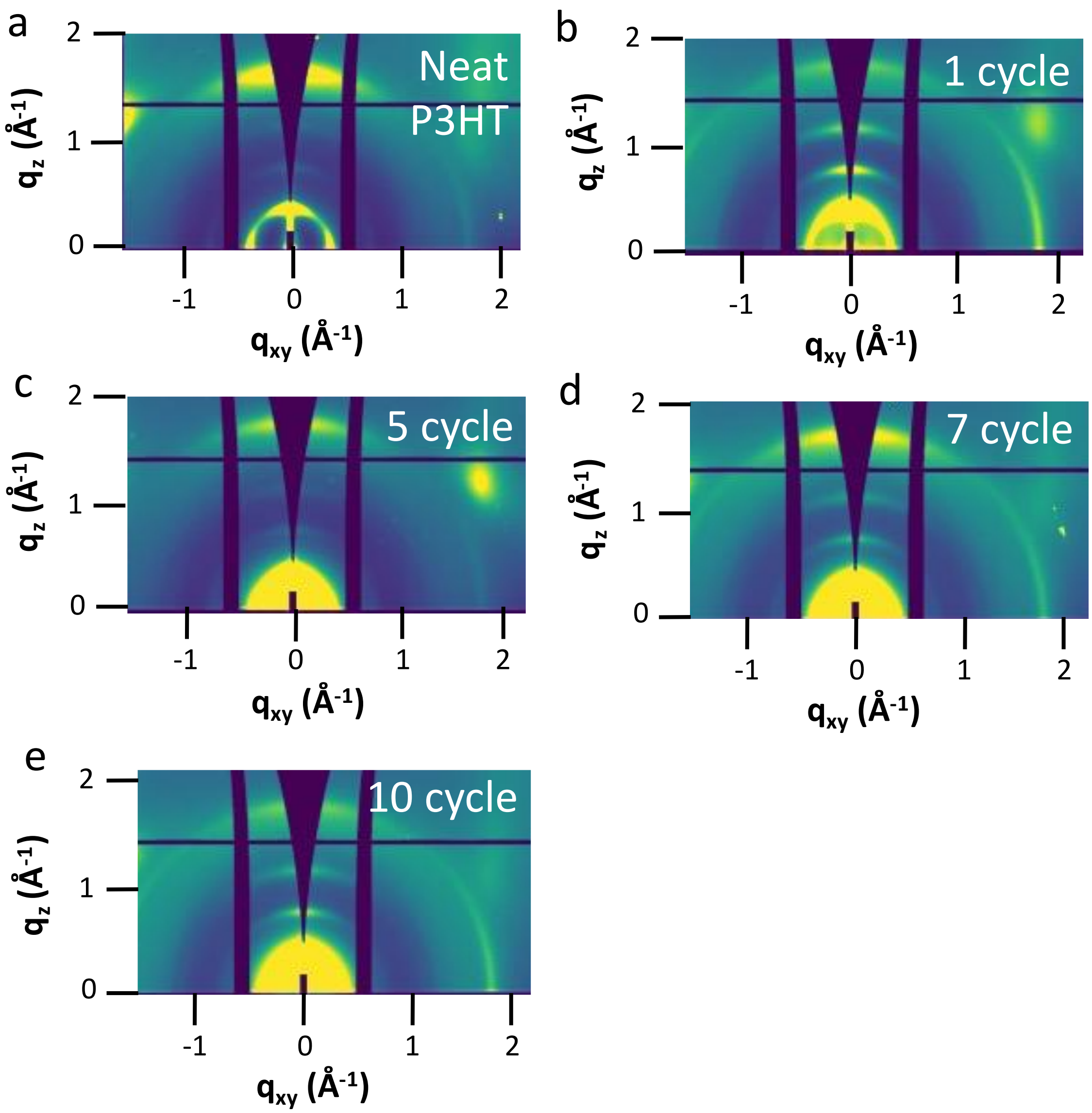

Figure S8. *GIWAXS diffractograms for (a) neat P3HT and P3HT exposed to (b) 1, (c) 5 , (d) 7 and (e) 10 cycles spray casted onto p-doped silicon wafers.*

Figures S6, S7 and S8 show GIWAXS scans of neat P3HT and VPI treated P3HT-TiO_x. The *d*-spacing between alkyl side chains (100 peaks) increases (*q*-spacing decreases) while the *d*-spacing between π-π stacks (010 peaks) decreases (*q*-spacing increases) for the infiltrated samples as compared to the neat. These structural changes indicate that as the $TiCl_4$ diffuses through the sample it does not disrupt much of the π-π stacking of the P3HT likely because the densely packed rigid backbone with strong π-π intermolecular forces leads to poor diffusion. On the other hand, the higher free volume and more mobile alkyl side chains likely lead to increased diffusion through the lamellar space, leading the resultant TiO_x clusters to entrap themselves amongst the alkyl side chains. The same trends in structural changes have been observed for liquid doping of P3HT with

Fe dopants, and the most common conclusion drawn from the GIWAXS data is that the counterions used in the dopant intercalate in between the alkyl chains and not the π-π stacks.[4,5] The conclusions made in these other studies and the GIWAXS data measured herein indicate that $TiCl_4$ primarily diffuses through the alkyl chains.

Additionally, VPI seems to have an effect on the crystalline orientation of the P3HT polymer. This can be seen in Figure S8 as the band at $q_z = 1.8$ Å^{-1} seems to become more in-plane (ring-like) and less out-of-plane. This is indicative of the polymer changing from face-on to a mix of face-on and edge-on orientation.[6]

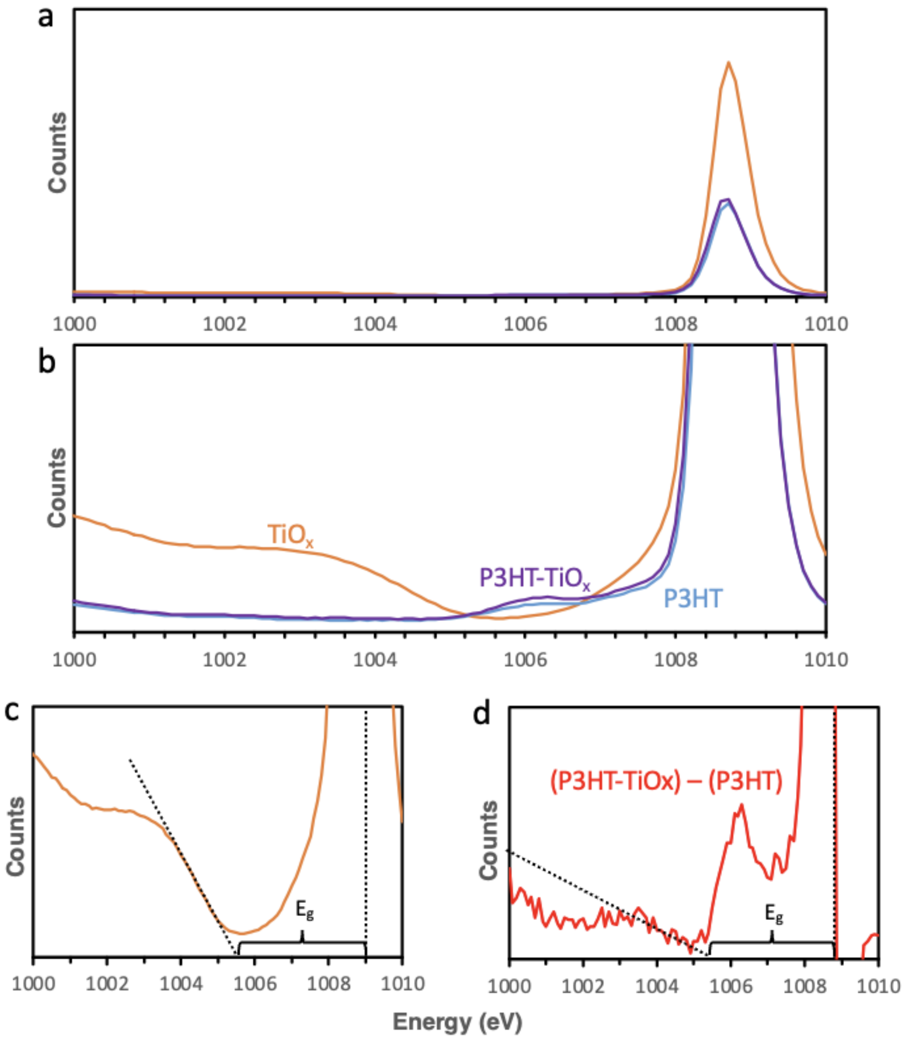

Figure S9. *Reflective Electron Energy Loss Spectrum (REELS) Spectra of (a) and (b) neat P3HT, ALD-deposited TiO_x, and VPI synthesized P3HT-TiOx. Bandgap of the (c) pure TiO_x film and the (d) TiO_x in the hybrid P3HT-TiO_x determined using the difference between the P3HT-TiOx and neat P3HT spectra.*

Figure S9 shows the REELS spectra used to determine the bandgap of the inorganic TiO_x in both its pure form and the clusters within the P3HT-TiO_x hybrid material. The bandgap is the difference between the incident beam (1008.7 eV) and the x-intercept of a linear line through the elastically scattered portion of curve. To obtain the bandgap of the TiO_x in the hybrid, the neat P3HT curve

was subtracted from the P3HT-TiO_x hybrid curve, as shown in Figure S9. The bandgap for pure TiO_x was found to be 3.17 eV and for that in the hybrid it was 3.05eV, though there is considerably more error in the hybrid because of its relatively low fraction of Ti. The similar band gaps give us indication that there is minimal electron orbital interaction between the infiltrated inorganic and organic in the ground state. Furthermore, since it is generally established that low temperature (<150°C) ALD deposition of TiO_2 from $TiCl_4$ and H_2O results in amorphous films[7,8] and the infiltrated inorganics show a similar band gap to an amorphous TiO_2, we believe the infiltrated inorganics are amorphous in structure. Due to the small length scale of the infiltrated inorganics, as shown in Figure S4, there is minimal long-range order in their structure. This makes direct probing of the crystalline state extremely difficult. However, based on the evidence presented and knowledge of vapor deposition techniques, we believe it is reasonable to conclude that the infiltrated inorganics are amorphous.

S5. Exciton Quenching Mechanism

If there were orbital mixing in the ground (non-excited) state, we would expect to see new peaks in the UV-Vis spectra. The only new peaks we see in the UV-Vis spectra are the polaronic peak and the metal oxide absorption. We know that these absorption are due to changes in the P3HT electronic structure and existence of the metal oxide, respectively. Therefore, the TiO_x and P3HT orbitals do not interact in the ground state. This narrows down the type of quenching to either collisional quenching or resonance energy transfer (RET). Isolating the exact mechanism is difficult. The primary difference is that collisional quenching requires a much smaller distance (a few angstroms) between quencher and fluorophore than RET.[9] However, the exciton diffusion length, which helps determine collisional quenching, is much larger for P3HT than the molecules these models were developed to explain. Regardless of the exact mechanism, this PL quenching strongly implies that photoexcited electrons in the P3HT are being injected into the TiO_x inorganic and will support the photocatalysis proposed in Figure 1b.

S6. Measuring Photocatalytic Performance

Here we provide an example of how photocatalytic rate was calculated. This example data is collected from a pure TiO_2 ALD-deposited film on glass. Here, the film is exposed to broad band light while submerged in a solution of 0.0004 wt% MB, as depicted in Figure 5a. Figure S10a plots a series of absorbance spectra taken from the MB solution at varying times under constant illumination. Figure S10b plots the peak absorbance from these UV-Vis spectra as a function of time. Figure S10c plots the natural log of these absorbances normalized to the initial absorbance (after 30 min) versus time to linearize the data according to a first-order rate law equation:

$$\ln(A_t/A_0) = k * t[\text{min}] \quad \text{Eq. S1}$$

where A_t = absorbance at time t, A_0 = initial absorbance (after 30 mins), k = rate constant and t = time in minutes. The slope of this linear fit is equal to the rate constant for this photocatalytic degradation reaction. Finally, to normalize for any slight variations in sample size, we weigh the slide and correlate it to the surface area using equation 2, where we assume the polymer contributes a negligible amount of mass.

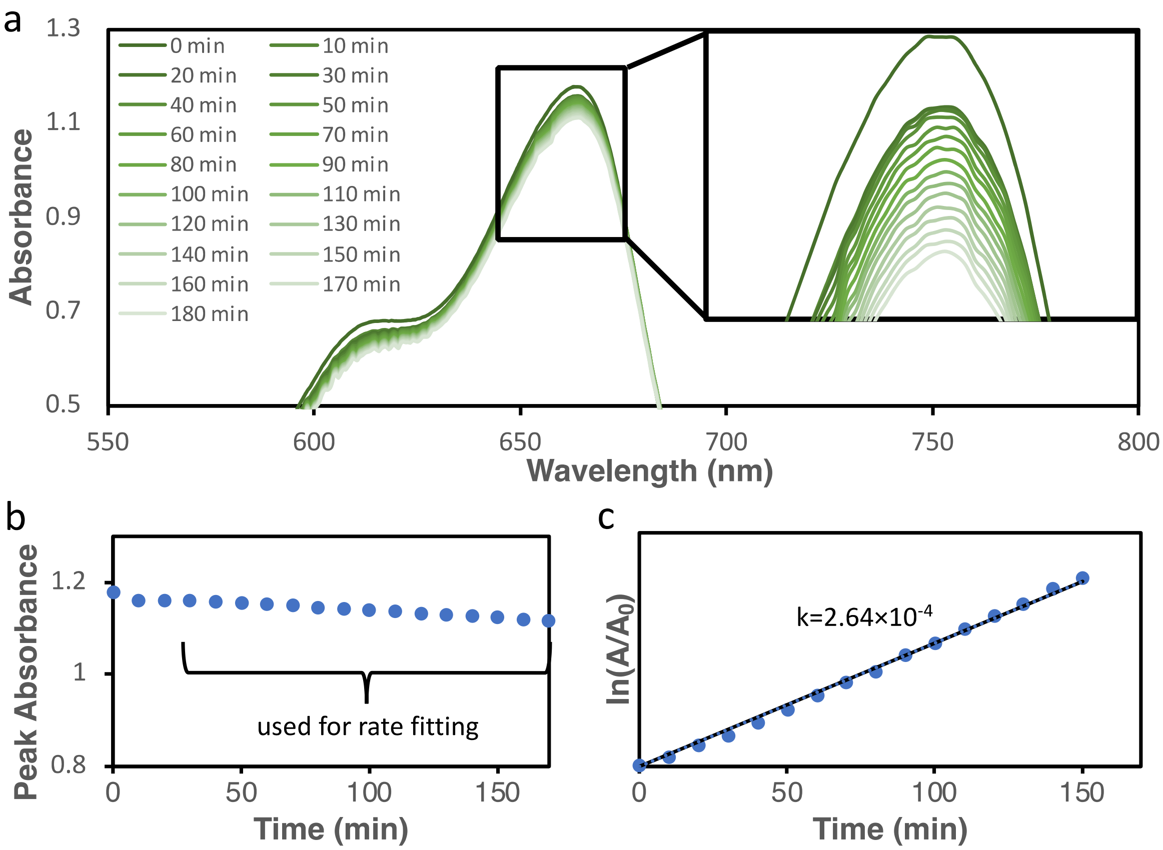

Figure S10. *Example of how photocatalytic degradation rate is determined using UV-Vis spectroscopy for a control system of 50-cycle (~2.8 nm) ALD-deposited TiO_2 film on glass. (a) UV-Vis spectra collected from a methyl blue solution containing an immersed TiO_x photocatalyst and exposed to broadband light for varying time intervals; inset enlarges the peak absorbance. (b) Plot of the peak UV-Vis absorbance of the methyl blue solution over time with the latter 150 mins used for rate fitting. (c) Fit made to absorbance data using the first order rate law equation to obtain a k-value.*

S7. Catalyst Architecture Considerations

The four key factors that have been identified for a high-performing CP-MO_x are the (1) photosensitivity, (2) metal-oxide-to-dye contact, (3) metal oxide surface area, and (4) the ability to inject excitons generated in P3HT into the metal oxide. The (4) exciton injection and (2) the need for metal-oxide-to-dye contact are particularly important to consider when designing the photocatalyst architecture. When the CP is illuminated and excitons are generated, only excitons generated “close enough” to the MO_x species will get injected into the inorganic catalyst. Therefore, only the MO_x clusters in direct contact with or near the CP are actually photosensitized. Furthermore, these metal oxide clusters must be near or at the chemical interface with the species intended for degradation (e.g., liquid dye solution). If the CP-MO_x photocatalysts are designed so that the CP is covering the MO_x there will be significant hinderance to the catalytic ability because the dye first needs to diffuse through the CP layer before it can reach the MO_x to be degraded. In other words, CP-MO_x photocatalysts should be designed so that the MO_x is near the surface.

Figure S11 presents a variety of architectures for CP-MO_x photocatalysts and provides qualitative assessments for each design’s effectiveness in achieving each of the critical design parameters. Architecture (e) is the main one studied in this publication. However, we have made test structures mimicking each of the other architectures to confirm their effects on limiting performance. The results for these un-optimized designs are presented in Figure S12. As can be seen, by adding a layer of CP onto either the TiO_x or the VPI synthesized P3HT-TiO_x, there is a significant reduction in the photocatalytic rate even below that of just TiO_x.

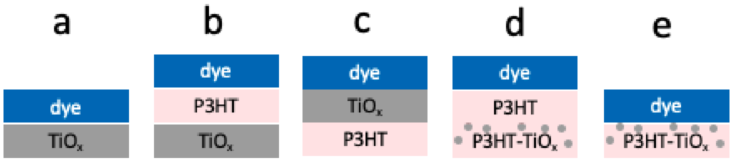

	a	b	c	d	e
Photosensitive	↓	↑	↑	↑	↑
TiO_x-dye contact	↑	↓	↑	↓	↑
Surface area	↓	↓	↓	↑	↑
Exciton Injection	↓	–	–	↑	↑

Figure S11. *Image depicting different photocatalyst designs during a dye degradation and their design efficacies (a)TiO_x (b)P3HT on TiO_x (c)TiO_x on P3HT and (d)P3HT with TiO_x clusters (e)P3HT with TiO_x clusters concentrated towards the surface.*

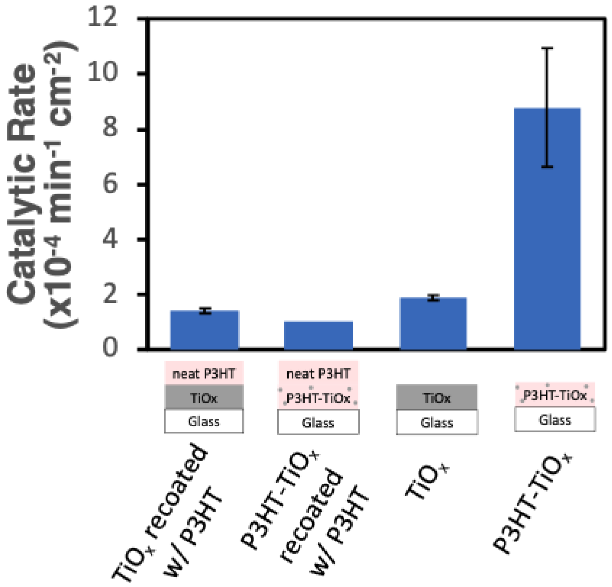

Figure S12. *Catalytic rates of (left to right) 50 cycles of ALD-deposited TiOx on glass recoated with ~150 nm P3HT, P3HT exposed to 5 cycles of VPI recoated with ~150 nm P3HT, 50 cycles of ALD-deposited TiOx on glass, and P3HT exposed to 5 cycles of VPI.*

With these key factors in mind, VPI is an excellent candidate for creating CP-MO_x photocatalysts. Having both a CP and MO_x means it is photosensitive. The MO_x is in good contact with the dye since the MO_x is concentrated towards the surface. The MO_x has a higher surface area because it forms small atomic clusters, as opposed to a smooth film. Finally, the atomic clusters mean a significant amount of MO_x atoms are in contact with the CP and are photosensitive.

S8. Comparison to Prior Reports

Table S1. *Comparison of other Conjugated Polymer-Metal Oxide Photocatalysts used for dye degradation from literature. Note: many studies were excluded from this comparison if the surface area for the catalyst could not be easily/confidently calculated.*

Catalyst Materials/Design	Surface Area Normalized k-value (min^{-1} cm^{-2})	Notes
This work	8.7×10^{-4}	
Polypyrrole grown onto ZnO_x Microrods[10]	3.72×10^{-4}	# of microrods/surface area of film was estimated at 20/100um^2 based on SEM images
Nanostructured TiO_2-polypyrrole composites[11]	1.69×10^{-4}	Synthesized polypyrrole on uncoordinated Ti sites leading to mostly monomer, dimer and trimer, meaning the polymer film is very thin. Diameter of composites was taken as the 250nm diameter of the polypyrrole granules, since they are much larger than the TiO_2 nanoparticles being used.
Polypyrrole grown onto TiO_2 nanoparticles[12]	2.09×10^{-5}	Methyl Orange was used instead of Methyl Blue but comparison was still made because TiO_2 was also used.
ZnO_x nanoparticles-polypyrrole composite[13]	1.12×10^{-6}	Surface area of composite not directly reported but reference [6] in article reports r=~175nm so this was used in calculations.
TiO_2 particles with Ag nanoparticles solution coated with P3HT[14]	1×10^{-6}	Methyl Orange was used instead of Methyl Blue but comparison was still made because P3HT and TiO_2 were also used
TiO_2 nanoparticles solution coated with polyaniline[15]	2.84×10^{-7}	
NiO particles synthesized *in situ* with polyaniline[16]	2.04×10^{-7}	Ni:monomer = ~1:54 mole ratio
ZnO_x nanoparticles solution coated with Polyaniline[17]	1.18×10^{-7}	

Table S1 shows details on our comparison with prior CP-MO_x photocatalysts made. It should be made clear that this is a general comparison and that normalizing all photocatalysis factors is essentially impossible.

S9. Hybrid Material Stability

Important to all catalyst materials is their stability. MO_x materials are well known for their stability, but CPs can degrade in air or water environments. For example, P3HT is known to degrade over time when exposed to oxygen and light.[18] To test the stability of the hybrid material made over the duration of the test period, FTIR and UV-Vis spectra (Figure S13a and S13b) of the samples were taken before and after submerging the hybrid film in water under illumination for 4 hours, the length of time it would be submerged in a catalysis measurement. To ensure removal of any sorbed water from the films, the samples were placed under a 125 Torr vacuum for 24 hours after submersion then analyzed using the appropriate spectroscopic technique. As seen in Figures 13a and b, the spectroscopic signatures of the hybrid film do not change significantly after water immersion and illumination. The UV-Vis spectra (Figure S13a) show the same absorption pattern before and after water submersion, indicating the electronic structure of the P3HT did not change. The minor difference in the amount of absorption can simply be due to slight differences in film thickness. FTIR spectra show the same functional groups present without any notable changes to peak positions, intensities or emergences of new peaks.

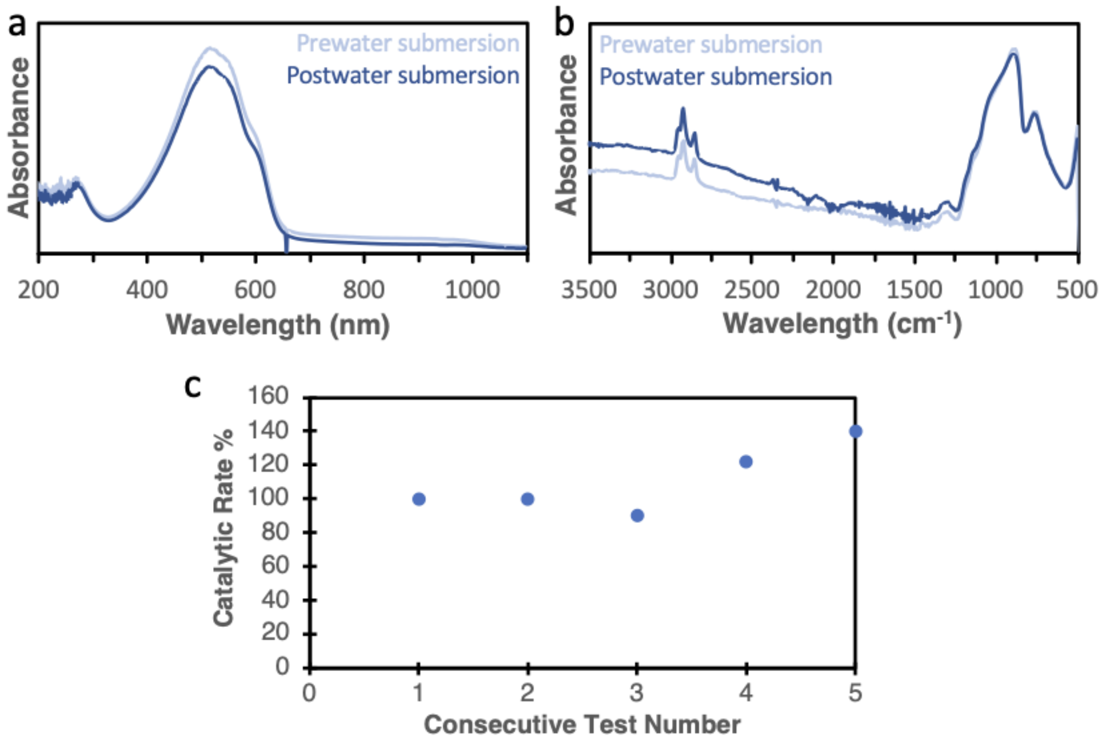

Figure S13. *UV-Vis (a) and FTIR (b) of P3HT exposed to 5 VPI cycle of* $TiCl_4$ + H_2O *before and after a 4 hour water submersion under illumination. Consecutive catalytic tests (c) of a P3HT-*TiO_x *hybrid film.*

As a final test to the stability of the hybrid catalyst, the same catalyst was subjected to multiple catalytic rate tests. As can be seen in Figure S13c, the catalytic rate of the sample measured remains relatively consistent through the 5 consecutive catalytic rate tests. Around the 4th catalytic test, the film begins to delaminate from the glass substrate, possibly artificially increasing the surface area which may explain the increase in catalytic rate. If further testing is to

be done, either an adhesion layer or a different casting method will need to be used. Regardless, initial tests show the hybrid is rather stable in the given test conditions and the catalyst is generally recyclable.

Of note, prior to the first catalytic test, the samples were presubmerged in a MB solution without illumination to allow for any reaction/sorption activities to occur, as described in the main manuscript. However, samples were not subjected to this presubmersion treatment after the first catalytic test, as it seemed reasonable that any reaction/sorption activities would have already occurred. The first 30 mins of absorption data collected was still ignored, as previously described in SI Section S6.

Although we have shown that the catalyst is stable for the measurement period studied, long-term stability issues for P3HT will still likely emerge over the course of weeks to months. Studies have been done to design solar cells where P3HT is stable even with light exposure.[19] Alternatively, more air-stable conjugated polymers have also been synthesized by both modifying the CP backbone or the side chains.[20,21] While we recognize these potential limitations for P3HT, its commercial availability and well-reported properties make it a good candidate for this initial demonstration.

References

(1) Lin, R.; Galili, M.; Quaade, U. J.; Brandbyge, M.; Bjørnholm, T.; Esposti, A. D.; Biscarini, F.; Stokbro, K. Spontaneous dissociation of a conjugated molecule on the Si(100) surface. *The Journal of Chemical Physics* **2002**, *117* (1), 321-330. DOI: 10.1063/1.1480857 (acccessed 7/30/2023).
(2) McGuinness, E. K.; Zhang, F.; Ma, Y.; Lively, R. P.; Losego, M. D. Vapor Phase Infiltration of Metal Oxides into Nanoporous Polymers for Organic Solvent Separation Membranes. *Chemistry of Materials* **2019**, *31* (15), 5509-5518. DOI: 10.1021/acs.chemmater.9b01141.
(3) Aarik, J.; Aidla, A.; Mändar, H.; Uustare, T. Atomic layer deposition of titanium dioxide from TiCl4 and H2O: investigation of growth mechanism. *Applied Surface Science* **2001**, *172* (1), 148-158. DOI: https://doi.org/10.1016/S0169-4332(00)00842-4.
(4) Al Kurdi, K.; Gregory, S. A.; Gordon, M. P.; Ponder Jr, J. F.; Atassi, A.; Rinehart, J. M.; Jones, A. L.; Urban, J. J.; Reynolds, J. R.; Barlow, S.; et al. Iron(III) Dopant Counterions Affect the Charge-Transport Properties of Poly(Thiophene) and Poly(Dialkoxythiophene) Derivatives. *ACS Applied Materials & Interfaces* **2022**, *14* (25), 29039-29051. DOI: 10.1021/acsami.2c03414.
(5) Lim, E.; Peterson, K. A.; Su, G. M.; Chabinyc, M. L. Thermoelectric Properties of Poly(3-hexylthiophene) (P3HT) Doped with 2,3,5,6-Tetrafluoro-7,7,8,8-tetracyanoquinodimethane (F4TCNQ) by Vapor-Phase Infiltration. *Chemistry of Materials* **2018**, *30* (3), 998-1010. DOI: 10.1021/acs.chemmater.7b04849.
(6) Aubry, T. J.; Ferreira, A. S.; Yee, P. Y.; Aguirre, J. C.; Hawks, S. A.; Fontana, M. T.; Schwartz, B. J.; Tolbert, S. H. Processing Methods for Obtaining a Face-On Crystalline Domain Orientation in Conjugated Polymer-Based Photovoltaics. *The Journal of Physical Chemistry C* **2018**, *122* (27), 15078-15089. DOI: 10.1021/acs.jpcc.8b02859.
(7) Niemelä, J.-P.; Marin, G.; Karppinen, M. Titanium dioxide thin films by atomic layer deposition: a review. *Semiconductor Science and Technology* **2017**, *32* (9), 093005. DOI: 10.1088/1361-6641/aa78ce.
(8) Piercy, B. D.; Leng, C. Z.; Losego, M. D. Variation in the density, optical polarizabilities, and crystallinity of TiO2 thin films deposited via atomic layer deposition from 38 to 150 °C using the titanium tetrachloride-water reaction. *Journal of Vacuum Science & Technology A* **2017**, *35* (3). DOI: 10.1116/1.4979047 (acccessed 4/29/2024).
(9) Jin, Z.; Gao, H.; Hu, L. Removal of Pb(ii) by nano-titanium oxide investigated by batch, XPS and model techniques. *RSC Advances* **2015**, *5* (107), 88520-88528, 10.1039/C5RA14004H. DOI: 10.1039/C5RA14004H.
(10) Yan, B.; Wang, Y.; Jiang, X.; Liu, K.; Guo, L. Flexible Photocatalytic Composite Film of ZnO-Microrods/Polypyrrole. *ACS Applied Materials & Interfaces* **2017**, *9* (34), 29113-29119. DOI: 10.1021/acsami.7b08462.
(11) Dimitrijevic, N. M.; Tepavcevic, S.; Liu, Y.; Rajh, T.; Silver, S. C.; Tiede, D. M. Nanostructured TiO2/Polypyrrole for Visible Light Photocatalysis. *The Journal of Physical Chemistry C* **2013**, *117* (30), 15540-15544. DOI: 10.1021/jp405562b.
(12) Li, S.; Chen, M.; He, L.; Xu, F.; Zhao, G. Preparation and characterization of polypyrrole/TiO2 nanocomposite and its photocatalytic activity under visible light irradiation. *Journal of Materials Research* **2009**, *24* (8), 2547-2554. DOI: 10.1557/jmr.2009.0316.

(13) Ovando-Medina, V. M.; López, R. G.; Castillo-Reyes, B. E.; Alonso-Dávila, P. A.; Martínez-Gutiérrez, H.; González-Ortega, O.; Farías-Cepeda, L. Composite of acicular rod-like ZnO nanoparticles and semiconducting polypyrrole photoactive under visible light irradiation for methylene blue dye photodegradation. *Colloid and Polymer Science* **2015**, *293* (12), 3459-3469. DOI: 10.1007/s00396-015-3717-2.
(14) Zhang, J.; Huang, Y.; Dan, Y.; Jiang, L. P3HT/Ag/TiO2 ternary photocatalyst with significantly enhanced activity under both visible light and ultraviolet irradiation. *Applied Surface Science* **2019**, *488*, 228-236. DOI: https://doi.org/10.1016/j.apsusc.2019.05.150.
(15) Zhang, H.; Zong, R.; Zhao, J.; Zhu, Y. Dramatic Visible Photocatalytic Degradation Performances Due to Synergetic Effect of TiO2 with PANI. *Environmental Science & Technology* **2008**, *42* (10), 3803-3807. DOI: 10.1021/es703037x.
(16) Vidya, J.; Balamurugan, P. Photocatalytic degradation of methylene blue using PANi—NiO nanocomposite under visible light irradiation. *Materials Research Express* **2019**, *6* (9), 0950c0958. DOI: 10.1088/2053-1591/ab34a3.
(17) Zhang, H.; Zong, R.; Zhu, Y. Photocorrosion Inhibition and Photoactivity Enhancement for Zinc Oxide via Hybridization with Monolayer Polyaniline. *The Journal of Physical Chemistry C* **2009**, *113* (11), 4605-4611. DOI: 10.1021/jp810748u.
(18) Yaghoobi Nia, N.; Bonomo, M.; Zendehdel, M.; Lamanna, E.; Desoky, M. M. H.; Paci, B.; Zurlo, F.; Generosi, A.; Barolo, C.; Viscardi, G.; et al. Impact of P3HT Regioregularity and Molecular Weight on the Efficiency and Stability of Perovskite Solar Cells. *ACS Sustainable Chemistry & Engineering* **2021**, *9* (14), 5061-5073. DOI: 10.1021/acssuschemeng.0c09015.
(19) Holliday, S.; Ashraf, R. S.; Wadsworth, A.; Baran, D.; Yousaf, S. A.; Nielsen, C. B.; Tan, C.-H.; Dimitrov, S. D.; Shang, Z.; Gasparini, N.; et al. High-efficiency and air-stable P3HT-based polymer solar cells with a new non-fullerene acceptor. *Nature Communications* **2016**, *7* (1), 11585. DOI: 10.1038/ncomms11585.
(20) Tournebize, A.; Gardette, J.-L.; Taviot-Guého, C.; Bégué, D.; Arnaud, M. A.; Dagron-Lartigau, C.; Medlej, H.; Hiorns, R. C.; Beaupré, S.; Leclerc, M.; et al. Is there a photostable conjugated polymer for efficient solar cells? *Polymer Degradation and Stability* **2015**, *112*, 175-184. DOI: https://doi.org/10.1016/j.polymdegradstab.2014.12.018.
(21) Shen, D. E.; Lang, A. W.; Collier, G. S.; Österholm, A. M.; Smith, E. M.; Tomlinson, A. L.; Reynolds, J. R. Enhancement of Photostability through Side Chain Tuning in Dioxythiophene-Based Conjugated Polymers. *Chemistry of Materials* **2022**, *34* (3), 1041-1051. DOI: 10.1021/acs.chemmater.1c03317.
